# Anaerobic Biohydrogenation of Isoprene by Acetobacterium wieringae Strain Y

**DOI:** 10.1128/mbio.02086-22

**Published:** 2022-11-07

**Authors:** Huijuan Jin, Xiuying Li, Hongyan Wang, Natalie L. Cápiro, Xiaocui Li, Frank E. Löffler, Jun Yan, Yi Yang

**Affiliations:** a Key Laboratory of Pollution Ecology and Environmental Engineering, Institute of Applied Ecology, Chinese Academy of Sciences, Shenyang, Liaoning, China; b University of Chinese Academy of Sciences, Beijing, China; c Department of Civil and Environmental Engineering, Auburn University, Auburn, Alabama, USA; d Center for Environmental Biotechnology, University of Tennessee, Knoxville, Tennessee, USA; e Department of Civil and Environmental Engineering, University of Tennessee, Knoxville, Tennessee, USA; f Department of Microbiology, University of Tennessee, Knoxville, Tennessee, USA; g Department of Biosystems Engineering & Soil Science, University of Tennessee, Knoxville, Tennessee, USA; h Biosciences Division, Oak Ridge National Laboratory, Oak Ridge, Tennessee, USA; McMaster University

**Keywords:** *Acetobacterium*, biohydrogenation, biotransformation, ene-reductase, isoprene

## Abstract

Isoprene is a ubiquitously distributed, biogenic, and climate-active organic compound. Microbial isoprene degradation in oxic environments is fairly well understood; however, studies exploring anaerobic isoprene metabolism remain scarce, with no isolates for study available. Here, we obtained an acetogenic isolate, designated Acetobacterium wieringae strain Y, which hydrogenated isoprene to a mixture of methyl-1-butenes at an overall rate of 288.8 ± 20.9 μM day^−1^ with concomitant acetate production at a rate of 478.4 ± 5.6 μM day^−1^. Physiological characterization demonstrated that isoprene was not utilized in a respiratory process; rather, isoprene promoted acetogenesis kinetically. Bioinformatic analysis and proteomics experiments revealed the expression of candidate ene-reductases responsible for isoprene biohydrogenation. Notably, the addition of isoprene to strain Y cultures stimulated the expression of proteins associated with the Wood-Ljungdahl pathway, indicating unresolved impacts of isoprene on carbon cycling and microbial ecology in anoxic environments (e.g., promoting CO_2_ plus H_2_ reductive acetogenesis while inhibiting methanogenesis). Our new findings advance understanding of microbial transformation of isoprene under anoxic conditions and suggest that anoxic environments are isoprene sinks.

## INTRODUCTION

Isoprene is a biogenically produced, volatile organic compound, and increased emissions progressively impact global climate (1–4). Isoprene is also a versatile monomer for polymer manufacturing and widely used in the chemical industry. Uncontrolled releases of isoprene in urban areas can cause detrimental effects on human health (e.g., irritation of the upper respiratory tract, isoprene-induced ozone formation in urban areas) (3, 5–7). Considering its impacts on climate, global carbon cycling, and human well-being, an exhaustive investigation into the microbial turnover (e.g., the underlying biotransformation and biodegradation mechanisms) of isoprene is warranted. Such information can improve global isoprene flux models, lead to better understanding of the fate, longevity, and environmental impacts of natural and anthropogenic isoprene, and assist the development of biotechnological solutions for mitigating isoprene emissions (1, 7).

Terrestrial and aquatic environments are important sources and sinks for isoprene, and various biological entities (e.g., plants, algae, bacteria) contribute to the production and utilization of isoprene (1, 8). The vast majority of isoprene is released by terrestrial plants, especially trees (8, 9). In addition to abiotic processes (e.g., air-ocean gas exchange driven by wind speed and temperature, photochemical oxidation) (10–12), bacterial processes are involved in isoprene transformation and degradation. Various bacterial isolates, predominantly of the phyla *Actinobacteriota* and *Proteobacteriota*, can utilize isoprene as a carbon and energy source under oxic conditions (1, 8). Genome analysis showed that actinobacterial isolates belonging to the genera *Rhodococcus*, Mycobacterium, and *Gordonia* all possess two linked operons (i.e., *isoABCDEF*, *isoGHIJ*) responsible for the stepwise conversion of isoprene via 1,2-epoxy-isoprene and a glutathione conjugate 1-hydroxy-2-glutathionyl-2-methyl-3-butene (8, 11, 13–16). Many aerobic isoprene-degrading microorganisms have been isolated from soil and the leaves of isoprene-producing trees (e.g., poplar, willow), including *Gordonia*, *Nocardia*, *Methylobacterium*, Pseudomonas, Klebsiella, *Alcaligenes*, *Arthrobacter*, *Variovorax*, *Ramlibacter*, *Sphingopyxis*, *Sphingobacterium*, *Sphingobium*, *Leifsonia*, *Micrococcus*, Mycobacterium, *Nocardioides*, *Loktanella*, *Shinella*, *Stappia*, *Pantoea*, *Bacillus*, and *Rhodococcus* strains (8), indicating that a diversity of aerobic bacteria contributes to isoprene turnover.

To date, few studies have reported on isoprene-degrading anaerobes, and isolates capable of isoprene degradation and transformation under anoxic conditions are not available. The contributions of anaerobic isoprene degradation and transformation are uncertain and possibly overlooked processes in isoprene cycling in the environment (8, 13). A prior study observed that methanogenic enrichment cultures containing Methanospirillum hungatei and Methanothrix soehngenii converted unsaturated hydrocarbons, including squalene and 1-hexadecene, to methane and carbon dioxide, but isoprene was not transformed (17). More recently, an acetogenic mixed culture dominated by *Acetobacterium* capable of reducing isoprene to a mixture of three methylbutene isomers (i.e., 2-methyl-1-butene, 3-methyl-1-butene, and 2-methyl-2-butene) in the presence of hydrogen and bicarbonate was reported (18). Physiological studies suggested that the *Acetobacterium* population in this mixed culture utilized isoprene as an electron acceptor for energy conservation under anoxic conditions (18). Axenic *Acetobacterium* cultures, such as A. woodii DSM 1030, A. malicum DSM 4132, and A. wieringae DSM 1911, failed to utilize isoprene, suggesting the ability to metabolize isoprene is not a shared feature among *Acetobacterium* spp. strains (18).

To advance the understanding of isoprene metabolism under anoxic conditions, sediment collected from a historically contaminated river was used as the source material to enrich anaerobes able to transform isoprene. Repeated transfers combined with dilution-to-extinction procedures in completely synthetic basal salt medium (19) amended with isoprene, hydrogen and bicarbonate yielded a novel Acetobacterium wieringae isolate, designated strain Y, capable of anaerobic isoprene reduction. Physiological experiments indicated that isoprene biohydrogenation was cometabolic not linked to energy conservation and growth. Proteomics experiments revealed key enzymes (e.g., candidate ene-reductases) responsible for isoprene biohydrogenation. Notably, isoprene stimulated the expression of proteins associated with the Wood-Ljungdahl pathway (WLP) in strain Y, indicating unresolved functions and impacts of isoprene on gene regulation and microbial ecology in anoxic environments.

## RESULTS

### Microbial transformation of isoprene.

**FIG 1:**
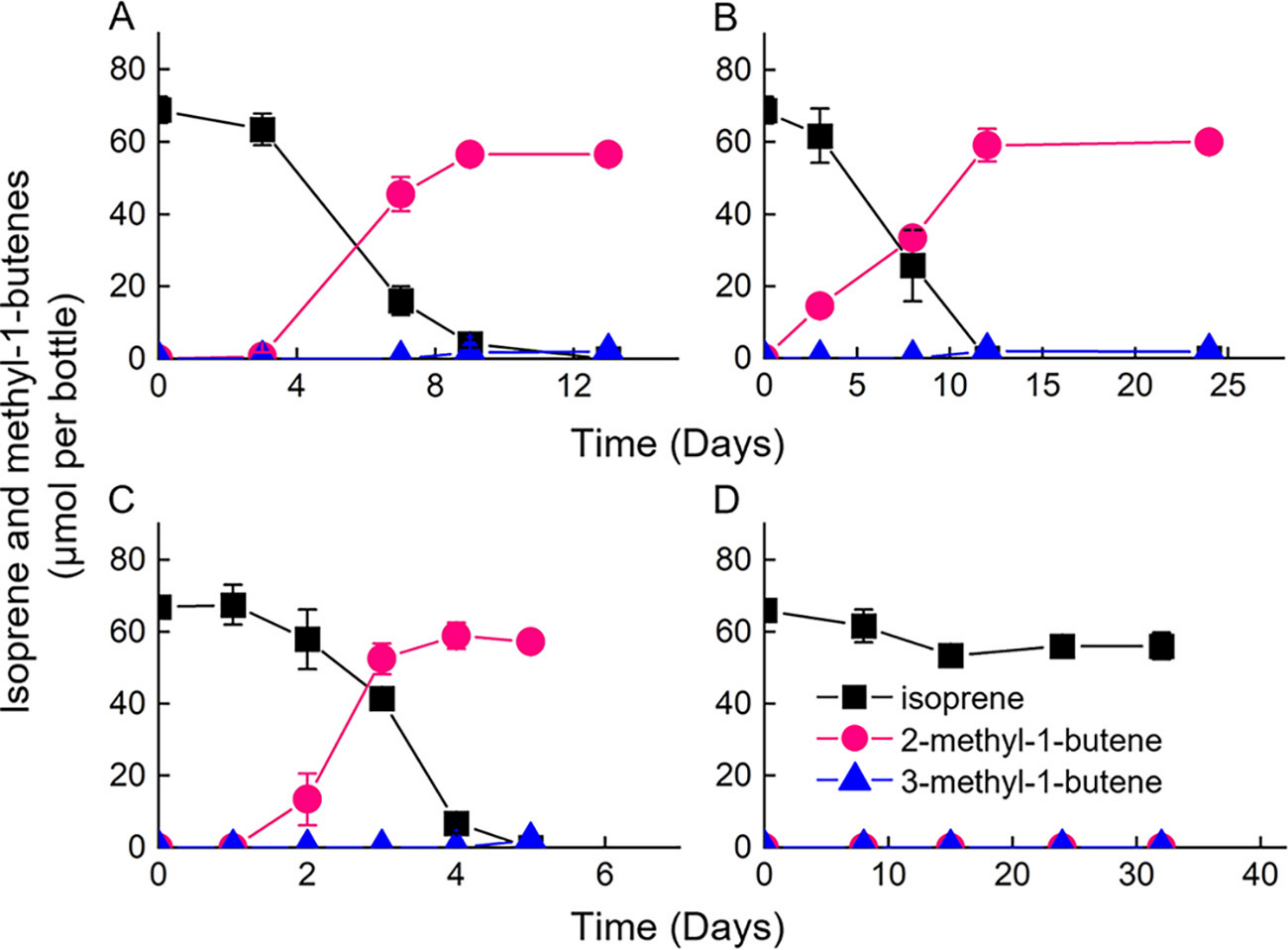
Isoprene consumption and transformation products formation in sediment microcosms (A), first-generation transfer cultures (B), eighth-generation transfer cultures (C), and abiotic control incubations with autoclaved sediment (D). Data shown represent the means ± standard deviations (*n* = 3), and error bars represent the standard deviations.

In Xi River sediment microcosms, the initial 68.8 ± 3.7 (mean ± standard deviation) μmol of isoprene completely disappeared with concomitant formation of 56.6 ± 2.7 μmol of 2-methyl-1-butene (~97%) and 2.0 ± 0.2 μmol of 3-methyl-1-butene (~3%) over a 13-day incubation period, indicating preferably biohydrogenation of the second C=C bond (i.e., positions 3 and 4) in isoprene (Fig. 1A). Isoprene transformation activity was maintained over consecutive transfers in solid-free enrichment cultures amended with lactate. For example, the first and eighth transfer cultures produced 61.7 ± 2.8 and 59.3 ± 2.8 μmol of methyl-1-butene isomers from 66.8 ± 2.8 μmol of isoprene, respectively. The isoprene hydrogenation rate of 262.3 ± 21.2 μM day^−1^ in the eighth transfer enrichment cultures was about 2.8-fold and 3.8-fold faster than that observed in the microcosms (94.2 ± 2.0 μM day^−1^) and the first transfer cultures (69.2 ± 9.9 μM day^−1^), respectively (Fig. 1B and C). Isoprene transformation was not observed in autoclaved control incubations (Fig. 1D). The substitution of lactate with acetate in ninth transfer enrichment cultures did not affect the rate or extent of isoprene hydrogenation (see Fig. S1A in the supplemental material). Taken together, these results demonstrated microbially mediated isoprene transformation in anaerobic enrichment cultures derived from river sediment.

10.1128/mbio.02086-22.1 FIG S1 (A) Biotransformation of isoprene in the ninth transfer of the enrichment cultures amended with acetate as carbon source. (B) Isoprene, methyl-1-butenes, and acetate in the HEPES-buffered strain Y cultures incubated for a week without H_2_ or without CO_2_/HCO_3_^−^, respectively. Download FIG S1, TIF file, 0.3 MB. Copyright © 2022 Jin et al. 2022 Jin et al. https://creativecommons.org/licenses/by/4.0/ This content is distributed under the terms of the Creative Commons Attribution 4.0 International license.

### Microbial community structures of the isoprene-transforming enrichment cultures.

**FIG 2:**
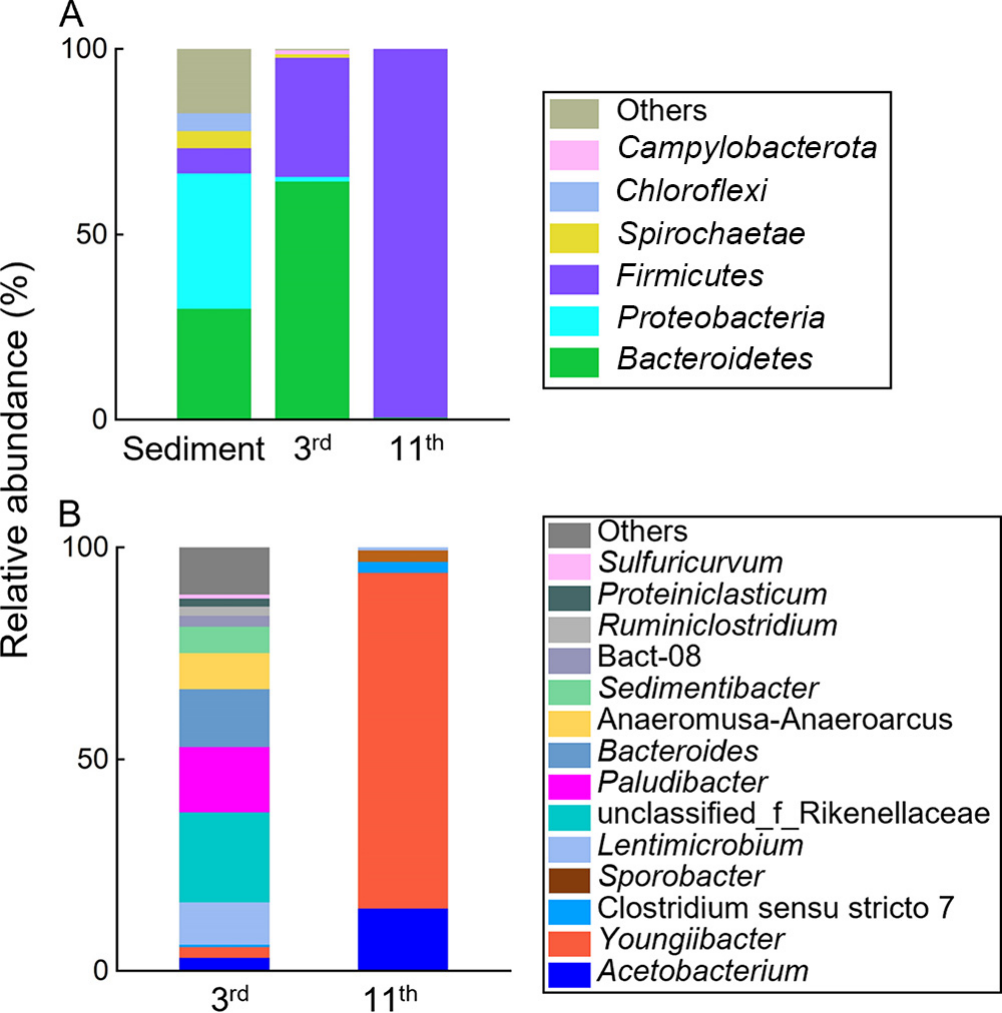
(A) Microbial compositions of the Xi River sediment sample, 3rd- and 11th-generation transfer cultures at the phylum level. (B) Microbial compositions of the 3rd- and 11th-generation transfer cultures at the genus level. The 3rd and the 11th transfer enrichments were cultivated with the mineral salt medium amended with lactate + hydrogen + isoprene and acetate + hydrogen + isoprene, respectively.

Amplicon sequencing of the 16S rRNA genes was applied to investigate the microbial population(s) responsible for anaerobic isoprene hydrogenation. Most of the sequences (i.e., up to 82.7%) obtained from the sediment microbiome were classified into five phyla, *Proteobacteria*, *Bacteroidota*, *Chloroflexi*, *Firmicutes*, and *Spirochaetota*, with relative abundances ranging from 4.7% to 36.6% (Fig. 2A). Time-series analysis revealed that members of *Bacteroidota* and *Firmicutes* were enriched in the isoprene-transforming enrichment cultures, increasing in abundances from 0.7 to 64.3% and from 32.2 to 99.3% of the total amplicon sequences, respectively. Among other detected phyla, only *Campylobacterota* sequences exceeded the 1% abundance. The most abundant genera in the third transfer of the enrichment culture amended with lactate were unclassified *Rikenellaceae* (21.2%), *Paludibacter* (15.4%), *Bacteroides* (13.7%), *Lentimicrobium* (10.0%), and *Acetobacterium* (3.2%). By comparison, the most abundant genera in the 11th transfer acetate-fed cultures were *Youngiibacter* (79.3%), *Acetobacterium* (14.8%), *Christensenellaceae* R-7 group (2.7%), and *Sporobacter* (2.6%) (Fig. 2B). The increased abundances of *Acetobacterium* in enrichment cultures suggested that *Acetobacterium* was the candidate population for anaerobic isoprene hydrogenation, consistent with a previous report (18).

### Isolation and characterization of an isoprene reducer.

**FIG 3:**
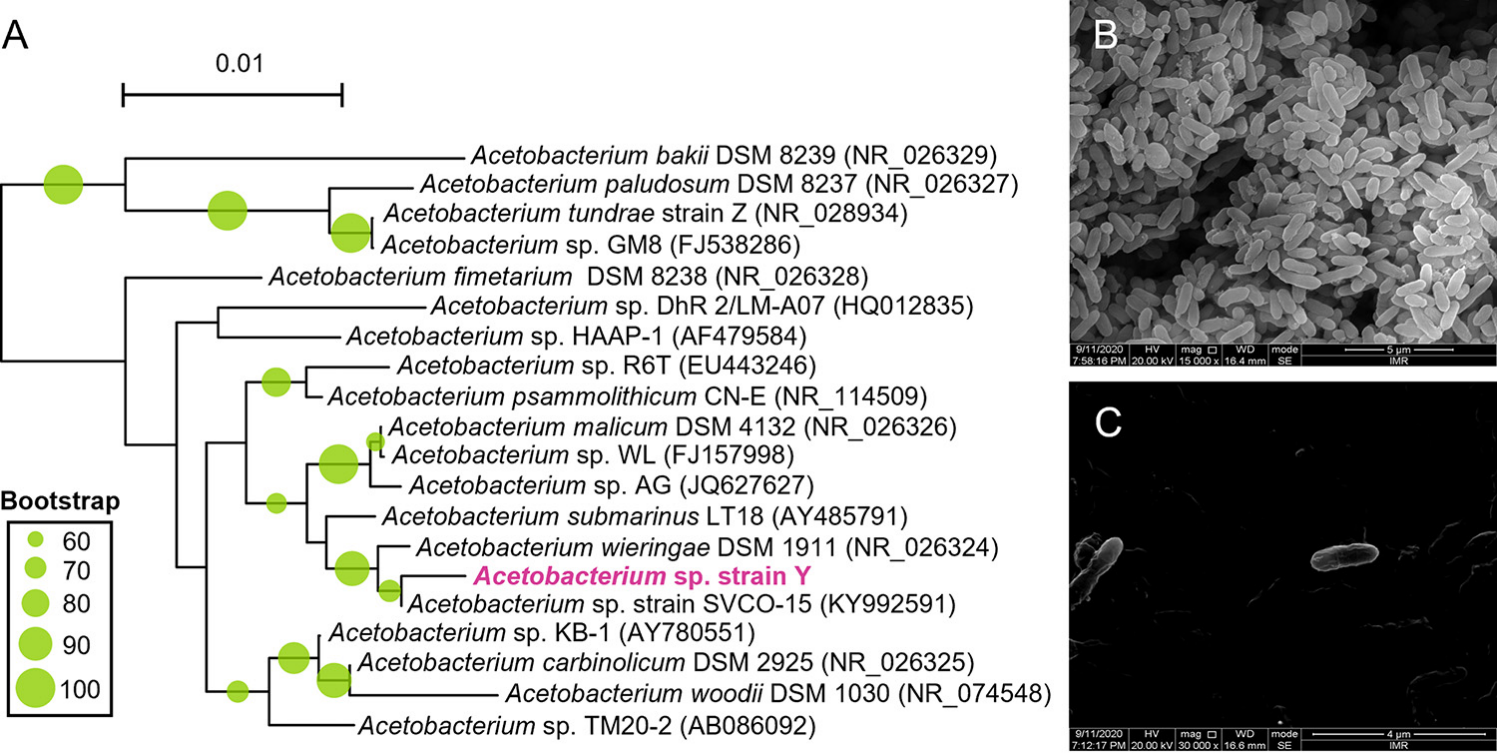
Phylogenetic identification and cell morphology of strain Y. (A) The 16S rRNA gene sequence-based phylogenetic tree was constructed with sequences derived from strain Y and closely related *Acetobacterium* sequences. (B and C) Scanning electron microscope micrographs showing the morphology of strain Y at ×15,000 (B) and ×30,000 (C) magnification. Scale bars, 0.01 substitutions per nucleotide position (A), 5 μm (B) and 4 μm (C).

Isolation of an isoprene reducer was achieved via six consecutive dilution-to-extinction efforts using the acetate-fed enrichment culture as an inoculum. This process yielded a pure culture designated as strain Y that reduced isoprene in hydrogen-amended bicarbonate-buffered mineral salt medium without additional organic carbon sources (e.g., lactate, acetate), and 16S rRNA gene amplicon sequencing revealed identical sequences belonging to *Acetobacterium* (Fig. S2). The closest relatives of strain Y are *A. wieringae* strain DSM 1911 (i.e., strain C) and *Acetobacterium* sp. strain SVCO-15, with 99.3% and 99.7% 16S rRNA gene sequence identities, respectively (Fig. 3A). Notably, *A. wieringae* strain DSM 1911 is unable to reduce isoprene (18, 20), suggesting strain Y represents a distinct *A. wieringae* strain. The purity of strain Y culture was further supported by the uniform cellular morphology observed in scanning electron microscopy (SEM) imaging. Strain Y cells were straight rods approximately 1 to 2 μm in length and 0.3 to 0.5 μm in diameter (Fig. 3B and C), in line with the previously described *Acetobacterium* isolates (e.g., *A. woodii* DSM 1030, *A. wieringae* DSM 1911, and *A. noterae* YOT-3T) (20–22). Spores were not observed in strain Y cultures. A putative pure culture containing a *Youngiibacter* sp. strain was also recovered from one of the dilution-to-extinction tubes; however, isoprene transformation did not occur in this *Youngiibacter* culture. Therefore, we concluded that this *Youngiibacter* sp. strain was not responsible for the observed isoprene transformation activity.

10.1128/mbio.02086-22.2 FIG S2 Examination of contamination in strain Y culture based on 16S rRNA gene amplicon sequencing. Isolation-1 and Isolation-6 indicate the first-round and sixth-round dilution-to-extinction isolation, respectively. All qualified 16S rRNA gene amplicon sequences derived from the sixth-round isolation were identical and belong to *Acetobacterium*. Download FIG S2, TIF file, 0.2 MB. Copyright © 2022 Jin et al. 2022 Jin et al. https://creativecommons.org/licenses/by/4.0/ This content is distributed under the terms of the Creative Commons Attribution 4.0 International license.

### Genomic features of strain Y.

To date, only few complete genomes have been available for the genus *Acetobacterium*, despite its indispensable roles for understanding the biochemistry and energy conservation in homoacetogens and potential applications for production of chemicals and fuels from carbon dioxide (21, 23). The complete circularized genome of strain Y has a size of 4,082,090 bp and a G+C content of 44.2 mol%. No plasmids were found. A total of 3,846 genes were annotated, consisting of 3,768 coding DNA sequences (CDSs), five 16S rRNA, six 5S rRNA, and five 23S rRNA genes, 58 tRNAs, and 4 ncRNAs (Fig. S3). Pairwise comparison of genome sequences performed with the Genome-to-Genome Distance Calculator found that strain Y and *A. wieringae* strain DSM 1911 shared 69.3 to 77.9% digital DNA-DNA hybridization, based on three different Genome BLAST Distance Phylogeny methods. ANIm, ANIb, and orthoANI analyses with JSpeciesWS and orthoANI demonstrated that the calculated average nucleotide identity (ANI) values for the genome pairwise comparison of strain Y and *A. wieringae* strain DSM 1911 were all above the 95% species delineation threshold (Table S1, Text S1) (24). Based on this information, we concluded that strain Y represents a novel strain of the species *A. wieringae*.

10.1128/mbio.02086-22.3 TEXT S1 Anaerobic biohydrogenation of isoprene by *Acetobacterium wieringae* strain Y. Download Text S1, DOCX file, 0.03 MB. Copyright © 2022 Jin et al. 2022 Jin et al. https://creativecommons.org/licenses/by/4.0/ This content is distributed under the terms of the Creative Commons Attribution 4.0 International license.

10.1128/mbio.02086-22.3 FIG S3 Circular genome map of *Acetobacterium wieringae* strain Y. Different tracks from outside to inside represent: (1) protein-coding genes on the forward strand (CDSs; yellow) interspersed with rRNA (red) and tRNA (blue) genes; (2) protein-coding genes on the reversed strand (CDSs; yellow) interspersed with rRNA (red) and tRNA (blue) genes; (3) GC content (black); (4) GC skew (green or purple denotes GC skew values greater than or less than the genome average, respectively); (5) size markers for the circular genome. Download FIG S3, TIF file, 0.1 MB. Copyright © 2022 Jin et al. 2022 Jin et al. https://creativecommons.org/licenses/by/4.0/ This content is distributed under the terms of the Creative Commons Attribution 4.0 International license.

10.1128/mbio.02086-22.8 TABLE S1 Genome statistics of 15 *Acetobacterium* strains. Download Table S1, DOCX file, 0.04 MB. Copyright © 2022 Jin et al. 2022 Jin et al. https://creativecommons.org/licenses/by/4.0/ This content is distributed under the terms of the Creative Commons Attribution 4.0 International license.

### Cometabolic transformation of isoprene by strain Y.

**FIG 4:**
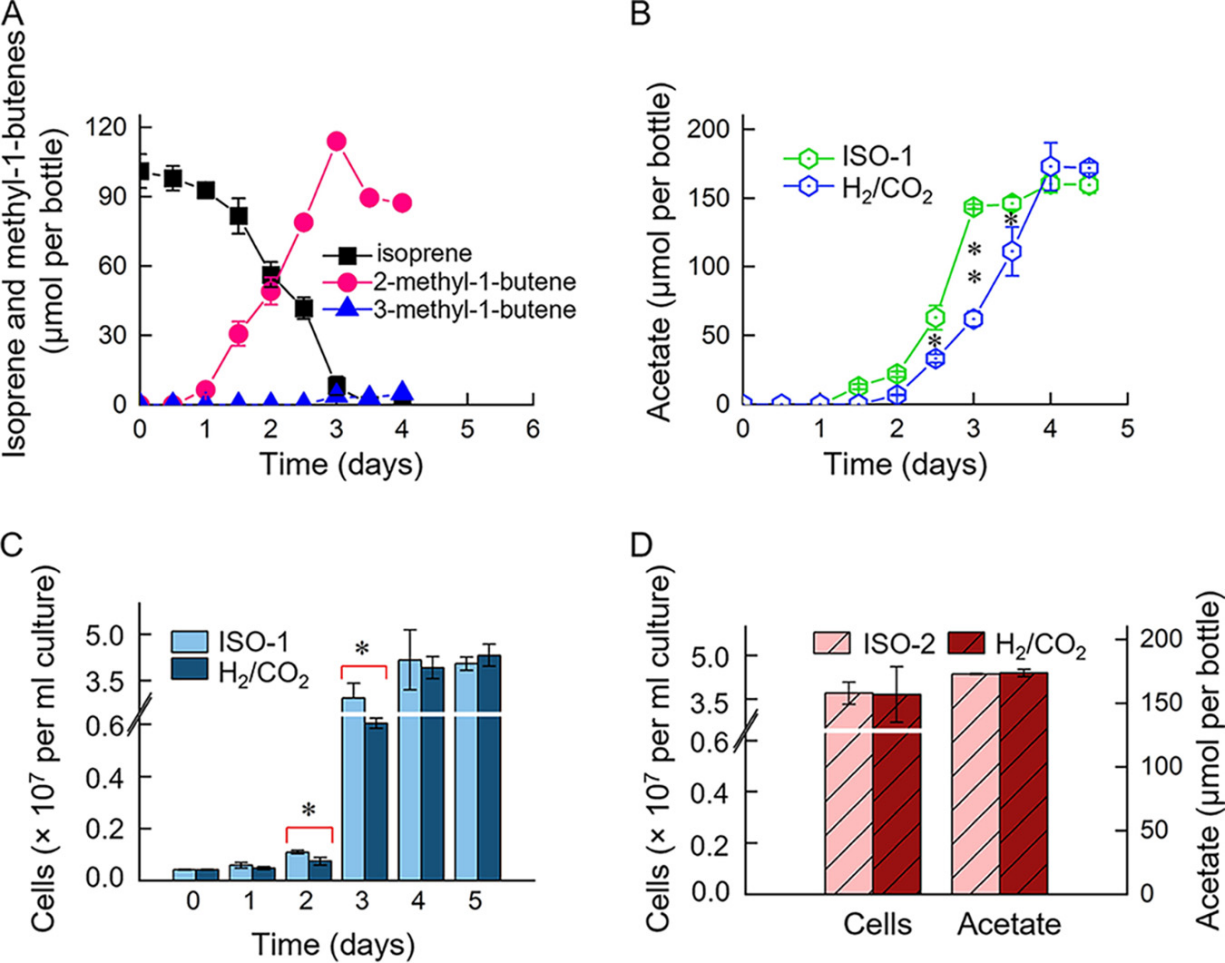
Isoprene reduction, acetate production, and biomass formation in isoprene-fed strain Y cultures amended with 20.0 mL H_2_ (ISO-1) or with (20.0 + 2.4) mL H_2_ (ISO-2; to completely hydrogenate 100.0 μmol of isoprene requires 2.4 mL H_2_, and the additional 20 mL H_2_ was to support reductive acetogenesis) or isoprene-free cultures amended with 20.0 mL H_2_ (H_2_/CO_2_ representing H_2_ plus CO_2_). (A) Isoprene reduction by strain Y in ISO-1 cultures. (B) Acetate formation in ISO-1 and H_2_ plus CO_2_ cultures. (C) Cell numbers in ISO-1 and H_2_ plus CO_2_ cultures. (D) Cell numbers and acetate at the end of incubation in ISO-2 and H_2_ plus CO_2_ cultures. All experiments were performed in triplicate and error bars represent one standard deviation. *, *P* < 0.05; **, *P* < 0.01.

Experiments were conducted to determine whether strain Y was able to conserve energy from isoprene reduction and to investigate whether other unsaturated hydrocarbons could be transformed by strain Y in bicarbonate-buffered medium. In strain Y cultures amended with isoprene and 20 mL H_2_ (ISO-1), 101.1 ± 7.3 μmol of isoprene was completely hydrogenated to 87.3 ± 1.3 μmol of 2-methyl-1-butene and 5.0 ± 0.6 μmol of 3-methyl-1-butene within 4 days, with concomitant production of 160.2 ± 6.2 μmol of acetate (i.e., acetogenesis from hydrogen and bicarbonate) (Fig. 4A and B). Acetate was also produced via acetogenesis in cultures without isoprene amendment, reaching the highest level of 172.8 ± 17.3 μmol over a 4-day incubation period (Fig. 4B). Acetate production in the isoprene-fed cultures was consistently about 10% less than that in cultures without isoprene amendment. Accompanying the formation of acetate, strain Y cell numbers in cultures with and without isoprene amendment increased from (4.15 ± 0.17) × 10^5^ to (4.05 ± 0.22) × 10^7^ cells mL^−1^ (97.6-fold increase) and (4.32 ± 0.36) × 10^7^ cells mL^−1^ (104.1-fold increase), respectively (Fig. 4C). During the first 3 days of incubation, the rates of acetate production from H_2_ and CO_2_/HCO_3_^−^ and biomass formation were significantly faster in cultures amended with isoprene (i.e., 478.4 ± 5.6 versus 207.3 ± 18.2 μM day^−1^; [9.69 ± 1.6] × 10^6^ versus [1.88 ± 0.06] × 10^6^ cells mL^−1^ day^−1^ in cultures with and without isoprene, respectively), indicating that isoprene impacted growth and acetate production from H_2_ and CO_2_/HCO_3_^−^ (Fig. 4B and C). Neither isoprene reduction nor acetate formation occurred in HEPES-buffered incubations in the absence of H_2_ or CO_2_/HCO_3_^−^ (Fig. S1B). These observations indicated that isoprene reduction to methyl-1-butenes depends on reductive acetogenesis from H_2_ and CO_2_/HCO_3_^−^ by strain Y, and both processes require the presence of CO_2_ and H_2_. The growth yield measurements indicated that strain Y cannot conserve energy from isoprene reduction but cometabolizes isoprene via a yet-to-be-identified enzyme system(s) expressed during H_2_ plus CO_2_ reductive acetogenesis. When CO_2_/HCO_3_^−^ was not limiting reductive acetogenesis, a mass balance calculation indicated that ~2.4 mL of H_2_ (~107.1 μmol) was consumed to hydrogenate 10 μL of isoprene (~100.0 μmol) according to equation 1 and equation 2, and the remaining H_2_ (i.e., ~17.6 mL) was used for acetogenesis via the Wood-Ljungdahl pathway, based on equation 3.

(1) $${\text{CH}}_{2}\text{=C}\left({\text{CH}}_{\text{3}}\right){\text{–CH=CH}}_{\text{2}}{+\text{H}}_{\text{2}}\to {\text{CH}}_{2}\text{=C}\left({\text{CH}}_{\text{3}}\right)\text{–}{\text{CH}}_{\text{2}}{\text{–CH}}_{\text{3, }}\;\Delta G^{0}=-132\text{ kJ/mol}$$

(2) $${\text{CH}}_{2}\text{=C }\left({\text{CH}}_{\text{3}}\right){\text{–CH=CH}}_{\text{2}}{+\text{H}}_{\text{2}}\to {\text{CH}}_{3}\text{–CH}\left({\text{CH}}_{\text{3}}\right)\text{–}\text{CH}{\text{=CH}}_{\text{2, }}\;\Delta G^{0}=-122\text{ kJ/mol}$$

(3) $$4{\text{ H}}_{2}+2{\text{ CO}}_{2}\to {\text{CH}}_{\text{3}}\text{COOH}+2{\text{ H}}_{\text{2}}\text{O, }\Delta G^{0}=\;-95\text{ kJ/mol}$$

Additional experiments were performed to confirm cometabolic transformation of isoprene. Similar cell densities ([3.71 ± 0.38] × 10^7^ versus [3.66 ± 0.96] × 10^7^ cells mL^−1^) and acetate production (172.8 ± 0.3 μmol versus 173.7 ± 2.8 μmol) were measured in the isoprene-amended (≈100.0 μmol) cultures with 20.0 + 2.4 mL H_2_ and the isoprene-free cultures with 20.0 mL H_2_ (Fig. 4D). The results suggested that the extra amount of 2.4 mL H_2_ (i.e., 107.1 μmol) in the isoprene-amended cultures was used to hydrogenate 99.4 ± 4.9 μmol of isoprene in a cometabolic manner.

**FIG 5:**
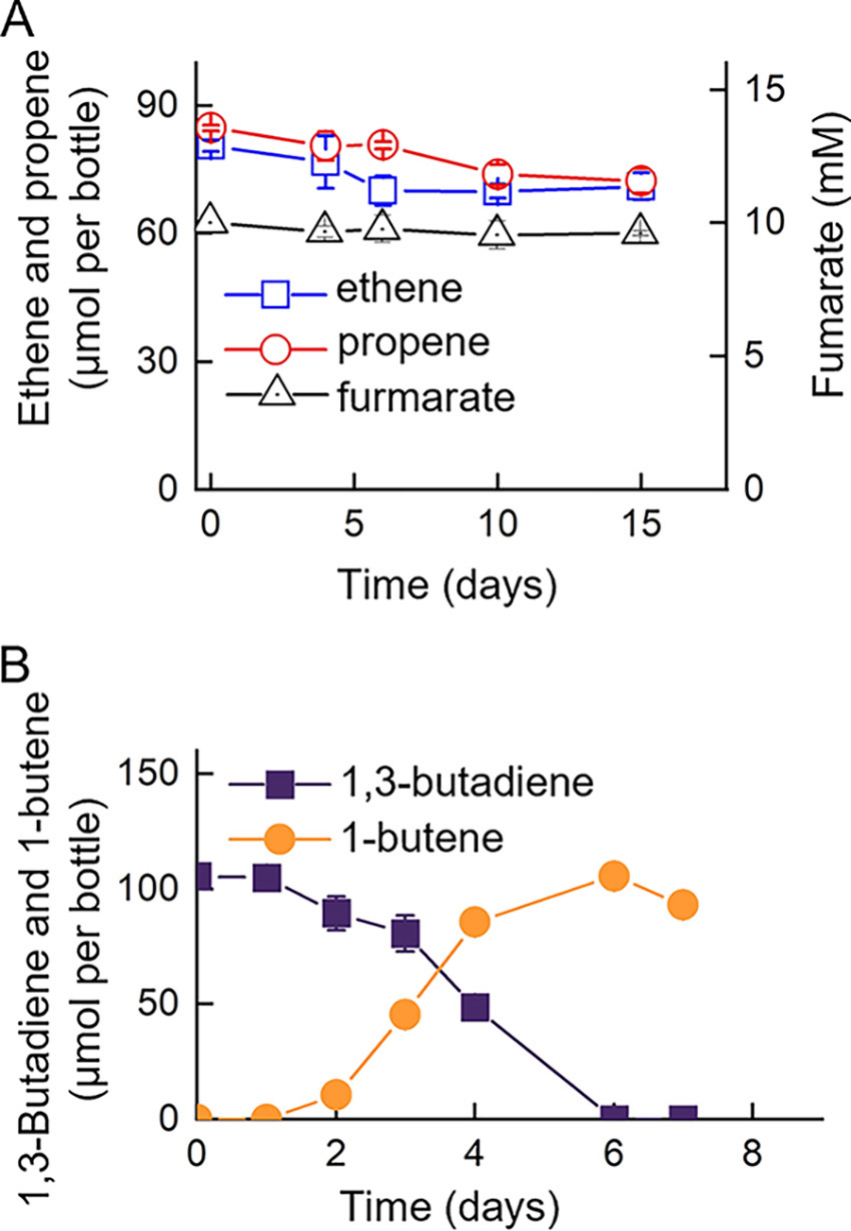
Anaerobic transformation of different ene compounds by strain Y. (A) Lack of biotransformation activity in cultures amended with ethene, propene or fumarate that contain a single C=C bond. (B) Transformation to 1,3-butadiene with two C=C bonds. All experiments were performed in triplicate and error bars represent one standard deviation.

Hydrogenation of a carbon-carbon double bond was not observed in ethene-, propene- or fumarate-amended strain Y cultures after a 15-day incubation period (Fig. 5A). In contrast, strain Y cultures completely transformed 105.6 ± 3.7 μmol of 1,3-butadiene to 93.1 ± 4.0 μmol of 1-butene within 7 days (Fig. 5B), indicating that strain Y selectively biohydrogenated one of the two double bonds in dienes (e.g., 1,3-butadiene, isoprene), but could not transform unsaturated hydrocarbons with a single C=C bond.

### Presence of putative ene-reductase (ER) genes in strain Y.

**FIG 6:**
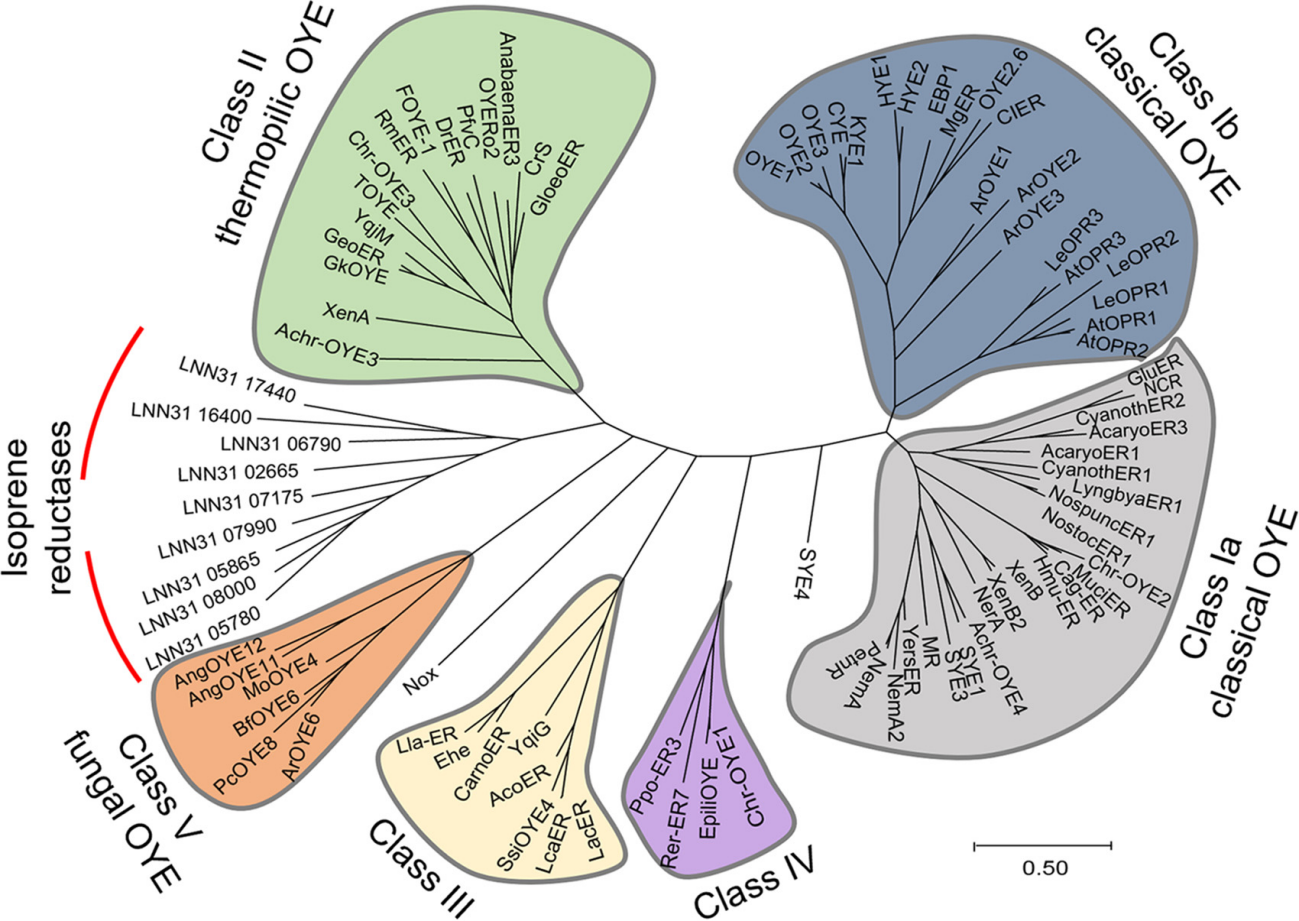
Phylogenetic tree constructed with nine putative novel OYEs (red lines) in *A. wieringae* strain Y and 89 Old Yellow Enzyme (OYE) sequences from fungal, plant, and bacterial origins. Twenty fungal OYEs (e.g., *Saccharomycotina*, Aspergillus niger) have been categorized into classical OYE clade Ib and clade V. Selected plant OYEs, including AtOPR1-3 from Arabidopsis thaliana and LeOPR1-3 from Solanum lycopersicum, were classified into the classic OYE clade Ib. Other OYE homologs originating from bacteria, including *Proteobacteria* (21%), *Actinobacteria* (4%), *Bacteroidetes* (8%), *Firmicutes* (18%), *Deinococcus-Thermus* (1%), and *Cyanobacteria* (10%), clustered separately in clade Ia, thermophilic-like clade II (thermostable OYE), clade III, and clade IV. The scale bar represents 0.05 substitutions per amino acid position.

The reduction of alkenes is catalyzed by a family of enzymes known collectively as ene-reductases (ERs) (25), and we hypothesized that ERs are responsible for catalyzing isoprene reduction in strain Y. A total of 44 putative ERs were annotated on strain Y genome, including 32 NAD(P)H/FAD-dependent oxidoreductases, 10 enzymes belonging to the salutaridine/menthone reductase-like subfamily of short-chain dehydrogenases and reductases (SDRs; EC 1.1.1.208) (26, 27), and two flavin reductases (see Data Set S1). Sequence similarity network (SSN) analysis (see Text S1) revealed that of the 32 NAD(P)H/FAD-dependent oxidoreductase proteins, 9 proteins clustered into the classical old yellow enzyme family (OYE; EC. 1.6.99.1), 11 proteins were distributed close to the OYE family in decentralized connections, and the remaining proteins (e.g., LNN31_03100) were dispersed outside the five ER groups due to low similarities. Five of 10 SDRs were closely related to the SDR family, while only one protein (LNN31_11860) was surrounded by the SDR enzymes, and two flavin reductases (LNN31_08725 and LNN31_17245) clustered closely with the quinone reductase-like ERs (QnoR) family (Fig. S4). We tentatively designated all putative ERs in strain Y as IsoR, standing for isoprene reductase. Phylogenetic analysis revealed that the nine OYE homologs of strain Y were separated from those in the well-characterized OYE branches (i.e., clade Ia and Ib, clade II, clade III, clade IV, and clade V) and formed a distinct new branch (Fig. 6). These OYE protein sequences shared 21.1 to 44.7% identities with previously characterized OYEs (Fig. 6; see also Data Set S2). Overall, these results indicated that strain Y possesses multiple putative ER-coding genes which have not been characterized, and their physiological and ecological function(s) remain to be elucidated.

10.1128/mbio.02086-22.4 FIG S4 The putative and characterized ene-reductases SSN generated with an alignment score threshold of 10^−10^. Different colors represent distinct ene-reductase clusters with similar biochemical activity. The putative ene-reductases found in *A. wieringae* strain Y are colored blue and designated as IsoR. OYE, Old Yellow Enzyme (purple); MDR, medium-chain dehydrogenase/reductase (green); EnoR, enoate reductase (light blue); SDR, short-chain dehydrogenase/reductase (pink); QnoR, quinone reductase-like ene-reductase (orange). Download FIG S4, TIF file, 0.8 MB. Copyright © 2022 Jin et al. 2022 Jin et al. https://creativecommons.org/licenses/by/4.0/ This content is distributed under the terms of the Creative Commons Attribution 4.0 International license.

### Comparative proteomic analysis identified isoprene-transforming ER(s).

**FIG 7:**
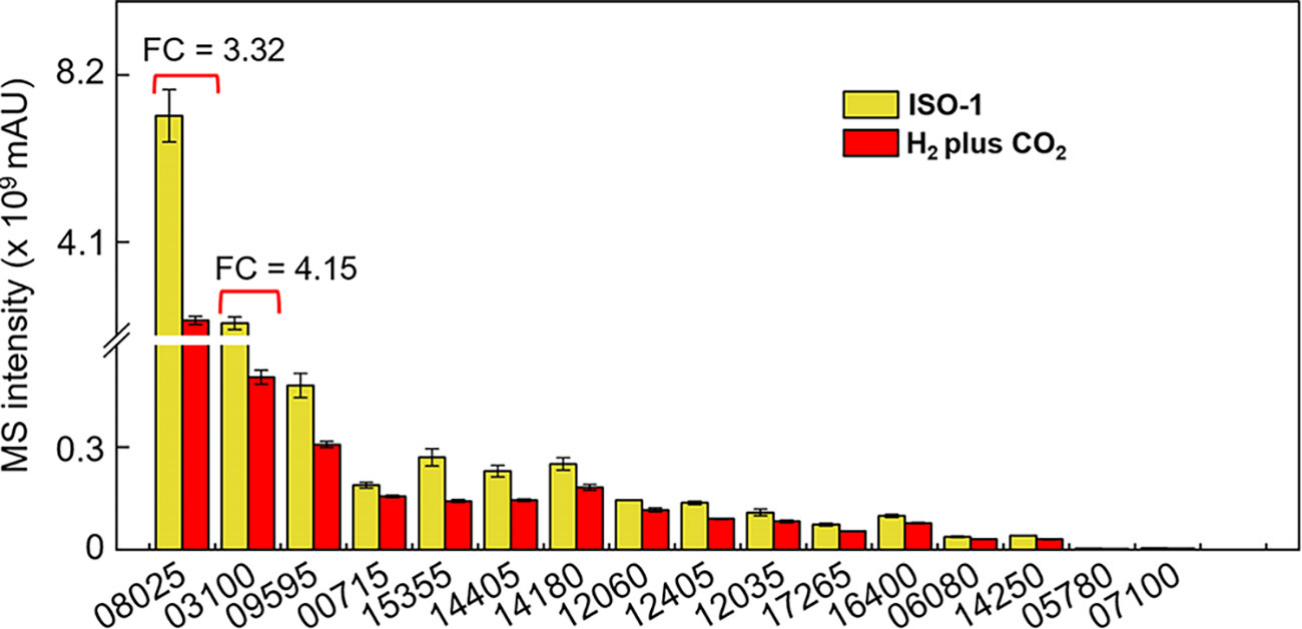
Abundances of expressed putative ene-reductases in isoprene-fed (ISO-1) cultures and cultures not receiving isoprene (H_2_ plus CO_2_). The numbers on the *x* axis represent the protein IDs (e.g., LNN31_08025). The listed putative ene-reductases had FC values >1.2, indicating significant differences in expression levels between the treatment and control groups. Error bars represent one standard deviation (*n* = 3).

To identify putative ER(s) catalyzing isoprene biohydrogenation in *A. wieringae* strain Y, proteomic analysis was performed with strain Y cultures grown in the H_2_-amended, bicarbonate-buffered mineral salt medium with or without isoprene. A total of 2,198 proteins were detected via 21,421 unique peptides. A total of 1,015 differentially expressed proteins were identified under the two different growth conditions, of which 540 were upregulated and 475 were downregulated in the isoprene-fed cultures when we used a fold change (FC) value of ≥1.2 in protein expression as a screening threshold for a physiologically significant change (Data Set S3). Seventeen putative ERs were upregulated, and the FAD-dependent oxidoreductase with protein ID LNN31_08025 (FC, >3.3; false-discovery rate [FDR], <0.002; mass spectometry [MS] intensity, [7.2 ± 0.6] × 10^9^) and the NAD(P)H-dependent oxidoreductase with protein ID LNN31_03100 (FC, >4.1; FDR, <0.002; MS intensity, [2.1 ± 0.2] × 10^9^) were abundantly expressed (Fig. 7). These two oxidoreductases shared low similarities to the OYE family enzymes catalyzing the reduction of unsaturated ketones, aldehydes, nitro alkenes, and carboxylic acids (Fig. S4) (28, 29). Other NAD(P)H/FAD-dependent oxidoreductases in isoprene-fed cultures showed only low abundances with an MS intensity of ≤(4.8 ± 0.4) × 10^8^ (Fig. 7). The putative ER LNN31_12060 belonging to the SDR family oxidoreductase was slightly upregulated (FC, 1.5; FDR, <0.002; MS intensity, [1.4 ± 0.1] × 10^8^) (Fig. 7).

The ER-like enzyme LNN31_08025 shared 100% amino acid identity to the FAD-dependent oxidoreductase (GenBank accession number WP_228882120.1) of the *A. wieringae* strain ISORED-2. Genes coding for the homologous proteins similar to the ER-like enzyme LNN31_08025 were also found in other sequenced *Acetobacterium* genomes. Another ER-like protein (LNN31_03100) exhibited 92 to 100% amino acid identities to the NAD(P)H-dependent oxidoreductases of *A. wieringae* strain ISORED-2, *Firmicutes* bacterium HGW-Firmicutes-17, *Acetobacterium* sp. strain KB-1, and *A. woodii* strain DSM 1030 (Fig. S5). Since the ability to reduce isoprene has not been reported in *A. woodii* strain DSM 1030 (18), the FAD-dependent oxidoreductase (LNN31_08025) is the candidate ER for catalyzing isoprene reduction to 2-methyl-1-butene and 3-methyl-1-butene isomers.

10.1128/mbio.02086-22.5 FIG S5 Phylogenetic tree of the putative ene-reductases, including LNN31_08025 (A) and LNN31_03100 (B). Trees were constructed with the neighbor-joining method using bootstrap resampling with 1,000 replications. Download FIG S5, TIF file, 0.5 MB. Copyright © 2022 Jin et al. 2022 Jin et al. https://creativecommons.org/licenses/by/4.0/ This content is distributed under the terms of the Creative Commons Attribution 4.0 International license.

### Isoprene affects the expression of Wood-Ljungdahl pathway genes in *A. wieringae* strain Y.

The Wood-Ljungdahl pathway (WLP) is the hallmark feature of *Acetobacterium* spp. and has been extensively investigated (30, 31). Similar to *A. woodii*, the complete WLP is divided into three separate gene clusters in *A. wieringae* strain Y. Cluster I is responsible for converting CO_2_ to formate and consists of genes encoding two formate dehydrogenases (Fdh1 and Fdh2) and accessory proteins. These genes were markedly divergent from those in the *A. woodii* strain DSM 1030 genome with low sequence identities ranging between 30.3 and 44.4%. Cluster II (i.e., the methyl branch of WLP that converts formate to methyl-tetrahydrofolate) consists of genes encoding formate tetrahydrofolate ligases (Fhs1 and Fhs2), cyclodeaminase/cyclohydrolase family protein (FchA), 5,10-methenyltetrahydrofolate cyclohydrolase (FolD), electron transport complex subunit RsxC (RnfC), methylenetetrahydrofolate reductase C-terminal domain-containing protein (MetV), and methylenetetrahydrofolate reductase (MetF) (Table 1). Cluster III, the carbonyl branch, contains genes encoding the CO dehydrogenase/acetyl-coenzyme A (CoA) synthase and methyltransferases (e.g., CooC1, AcsV, AcsA-E, and CooC2) for the conversion of methyl-tetrahydrofuran to acetyl-CoA. Unlike cluster I, cluster II and cluster III are highly conserved across all *Acetobacterium* genomes (Data Set S4). Interestingly, proteomic analysis revealed that WLP proteins, with the exception of Fdh, were expressed in higher abundances in the presence of isoprene. Twelve proteins encoded in cluster II and cluster III were differentially expressed when we compared the isoprene-amended and isoprene-free cultures. Specifically, 11 of these differentially expressed proteins (e.g., Fhs1, Fhs2, FchA, FolD, RnfC, MetV, MetF, AcsV, AcsA, CooC2, and AcsB) were significantly upregulated, but only one (AAA family ATPase, CooC1) was slightly downregulated in the presence of isoprene (Table 1). These results suggest that the presence of isoprene promotes the expression of genes associated with the WLP, and thus H_2_ plus CO_2_ reductive acetogenesis activity of strain Y. This observation is in line with the results of the physiological experiments (Fig. 4B and C).

**TABLE 1:** Expression of WLP-associated proteins^*a*^

Protein ID LNN31	WLP-associated enzyme	Expression ISO-1	Expression H_2_/CO_2_	FC	Regulated
00755	Formate dehydrogenase alpha (Fdh1)	ND	ND	ND	ND
03275	Formate dehydrogenase alpha (Fdh2)	ND	ND	ND	ND
15340	Formate-tetrahydrofolate ligase (Fhs1)	7.2 × 10^10^	2.8 × 10^10^	2.59	UP
15840	Formate-tetrahydrofolate ligase (Fhs2)	7.3 × 10^9^	2.5 × 10^9^	2.98	UP
15335	Cyclodeaminase/cyclohydrolase family protein (FchA)	4.2 × 10^9^	3.5 × 10^9^	1.22	UP
15330	5,10-Methenyltetrahydrofolate cyclohydrolase (FolD)	1.1 × 10^10^	7.8 × 10^9^	1.38	UP
15325	Electron transport complex subunit RsxC (RnfC)	1.1 × 10^10^	5.6 × 10^9^	1.88	UP
15320	Methylenetetrahydrofolate reductase C-terminal domain-containing protein (MetV)	1.1 × 10^9^	8.8 × 10^8^	1.24	UP
15315	Methylenetetrahydrofolate reductase (MetF)	5.3 × 10^9^	3.8 × 10^9^	1.38	UP
14595	AAA family ATPase (CooC1)	4.5 × 10^8^	5.5 × 10^8^	0.82	DOWN
14590	ASKHA domain-containing protein (AcsV)	3.4 × 10^8^	2.7 × 10^8^	1.23	UP
14575	Acetyl-CoA decarbonylase/synthase complex delta (AcsD)	7.2 × 10^9^	6.3 × 10^9^	1.14	NoSig
14570	Acetyl-CoA decarbonylase/synthase complex gamma (AcsC)	3.2 × 10^9^	2.8 × 10^10^	1.12	NoSig
14565	Dihydropteroate synthase (AcsE)	1.6 × 10^10^	1.5 × 10^10^	1.06	NoSig
14560	CO dehydrogenase catalytic subunit (AcsA)	1.9 × 10^10^	6.6 × 10^9^	2.83	UP
14555	AAA family ATPase (CooC2)	2.1 × 10^9^	1.7 × 10^9^	1.21	UP
14550	CO dehydrogenase/CO-methylating acetyl-CoA synthase complex (AcsB)	2.7 × 10^10^	1.4 × 10^10^	1.85	UP

a Differentially expressed protein selection was according to the following criterion: 0.83 < FC > 1.2. The protein expression numbers are average results of biological triplicates. ISO-1, the isoprene-fed treatment group; H_2_/CO_2_, the isoprene-free control group. FC, fold change; ND, not detected; UP, upregulated; DOWN, downregulated; NoSig, no significant differences.

## DISCUSSION

### A range of *Acetobacterium* spp. strains are implicated in anaerobic isoprene transformation.

The enrichment of *Acetobacterium* in isoprene-reducing mixed cultures suggested a role of *Acetobacterium* in the biotransformation of isoprene under anoxic conditions. Here, we isolated a novel Acetobacterium wieringae strain, designated as strain Y, and demonstrated that strain Y was able to reduce isoprene to 2-methyl-1-butene and 3-methyl-1-butene during growth with H_2_ and CO_2_/HCO_3_^−^ (Fig. S6). By comparison, Acetobacterium wieringae strain DSM 1911 was unable to reduce isoprene (20), while an *Acetobacterium*-containing enrichment culture derived from a sewage treatment plant in Sydney, Australia, could readily biotransform isoprene (18). Although not all *Acetobacterium* spp. can biotransform isoprene under anoxic conditions, Acetobacterium wieringae strains from distinct geographical locations share this capability, suggesting that *Acetobacterium* strains harboring ene-reductase(s) play a role in the global cycling of isoprene (10). The possibility that other microorganisms were also involved in isoprene reduction in the enrichment culture cannot be ruled out. For instance, members of the *Comamonadaceae* have been suggested to reduce isoprene (32, 33). Therefore, further research is warranted to explore the diversity of microorganisms capable of biotransforming isoprene under anoxic conditions.

10.1128/mbio.02086-22.6 FIG S6 Pathway for isoprene transformation in Acetobacterium wieringae strain Y. Download FIG S6, TIF file, 0.4 MB. Copyright © 2022 Jin et al. 2022 Jin et al. https://creativecommons.org/licenses/by/4.0/ This content is distributed under the terms of the Creative Commons Attribution 4.0 International license.

### Isoprene biohydrogenation is a cometabolic process.

Previous experiments suggested that an *Acetobacterium* population present in a mixed culture can utilize isoprene as electron acceptor, with lactate or molecular H_2_ as electron donor (18). Although isoprene reduction to methyl-1-butene is thermodynamically favorable, our results suggested that Acetobacterium wieringae strain Y does not utilize isoprene as a respiratory electron acceptor. When equal amounts of H_2_ (i.e., 20 mL) were provided, less acetate was produced in the cultures amended with isoprene than in those without isoprene addition. No differences in acetate production and growth yields were observed between strain Y cultivated with isoprene plus 22.4 mL hydrogen and strain Y grown only with 20 mL hydrogen without isoprene in the bicarbonate-buffered mineral salt medium. Although isoprene reduction did not exhibit energetic advantage, the presence of isoprene affects the kinetics of reductive acetogenesis and growth rates of Acetobacterium wieringae strain Y. Strain Y expressed WLP enzymes (e.g., Fhs, AcsB, AcsA, RnfC, FolD) in relatively higher abundances when exposed to isoprene, suggesting that the biohydrogenation of isoprene promotes the expression of enzymes involved in CO_2_ fixation. Isoprene, as a widely distributed and abundant natural product, has ecological implications and effects on anaerobic microbial processes. For instance, isoprene was found to moderately inhibit methanogenesis (18, 34). Homoacetogens are physiologically and thermodynamically less competitive for H_2_ than hydrogenotrophic methanogens; however, isoprene presence could change the dynamic ecological interactions of methanogens and homoacetogens by inhibiting methanogenesis and promoting H_2_ plus CO_2_ reductive acetogenesis kinetically.

### Biohydrogenation of isoprene by putative ene-reductases.

Strain Y selectively biohydrogenates and biotransforms conjugated diene compounds such as isoprene and 1,3-butadiene, but not compounds with a single double bond, such as ethene and propene. It has been well established that various enzyme families, collectively known as ene-reductases, can asymmetrically catalyze alkene reduction (28, 35, 36). A thorough annotation and search of the strain Y genome identified a total of 42 putative ERs; however, most of them could not be classified within the five previously defined ER classes (i.e., OYE, EnoR, SDR, MDR, QnoR). The high expression of an FAD-dependent oxidoreductase (LNN31_08025) suggested its involvement in the biohydrogenation of a specific double bond in some diene compounds. Future experiments including heterologous expression, site-directed mutagenesis, and molecular simulation should be performed to unravel the biochemical function(s) of these ERs. The diversity and distribution of ERs specifically targeting a single double bond in diene compounds will improve our understanding of the transport and environmental fate of isoprene and other diene compounds. ERs have potential industrial applications for the environmentally friendly production of fine chemicals, pharmaceuticals, and agrochemicals (25, 37). The novel ene-reductases identified in strain Y are promising catalysts for biotechnological applications, such as the efficient syngas fermentation for biofuel production.

## MATERIALS AND METHODS

### Chemicals.

Isoprene and 2-methyl-1-butene (both ≥99%) were purchased from Aladdin-Reagents Co., Ltd. (Shanghai, China). 3-Methyl-1-butene (≥95%) was purchased from Shanghai Macklin Biochemical Co., Ltd. (Shanghai, China). Ethene, propene, 1,3-butadiene, and 1-butene (all ≥99.7%) were purchased from Dalian Special Gases Co., Ltd. (Dalian, Liaoning, China). Low-melting-temperature agarose (gel strength, ≥200 g/cm^2^ for a 1% gel) was purchased from Sigma-Aldrich (St. Louis, MO, USA). All other chemicals were obtained from Macklin Co., Ltd. (Shanghai, China) or Sigma-Aldrich and were of analytical or higher grade.

### Microcosms and enrichment cultures.

Sediment samples were collected in July 2017 from the Xi River in Shenyang, Liaoning Province, China (41.6628°N, 123.1055°E). Unless otherwise specified, 160-mL glass serum bottles containing 100 mL of 30 mM bicarbonate-buffered (pH 7.2) mineral salt medium and a N_2_/CO_2_ headspace (80/20 [vol/vol]) (19) were used for cultivation. Triplicate microcosms were established with homogenized sediment slurries (~2 g) inside an anaerobic chamber (Coy Laboratory, Ann Arbor, MI). Each bottle received 5 mM lactate, 10 mL H_2_ (~414 μmol), Wolin vitamins (38), and 7 μL isoprene (~70.0 μmol). Isoprene, stored in the −20°C refrigerator before use, was added to the serum bottles using a 10-μL gas-tight Hamilton syringe (Hamilton Co., Reno, NV). Eight consecutive transfers (3% [vol/vol]) were performed under the same cultivation conditions to obtain enrichment cultures. In subsequent transfers, 5 mM acetate replaced lactate. All vessels were capped with autoclaved butyl rubber stoppers (Bellco Glass, Vineland, NJ, USA) and secured with aluminum crimps. Cultures prepared following the same procedure were autoclaved to serve as negative controls. All bottles were incubated at 30°C in the dark.

### Bacterial isolation and growth conditions.

For isolation, 2 μL of neat isoprene, Wolin vitamins, 1 mL of H_2_, and 5 mM acetate were added to 20-mL glass vials containing 9 mL of growth medium, 1% (wt/vol) low-melting-temperature agarose, and a N_2_/CO_2_ (80/20 [vol/vol]) headspace. A 1-mL aliquot of the ninth transfer of the isoprene-transforming enrichment culture served as the inoculum, which was serially diluted from 10^−1^ to 10^−12^. A white colony was picked up from the highest dilution tube showing complete conversion of isoprene to 2-methyl-1-butene and transferred to fresh liquid medium. This dilution-to-extinction procedure was repeated six times before culture purity was evaluated by SEM imaging, 16S rRNA gene amplicon sequencing, and Sanger sequencing.

Unless otherwise specified, the isolate, designated as strain Y, was routinely maintained in 160-mL serum bottles containing a N_2_/CO_2_ headspace (80/20 [vol/vol]) and 100 mL bicarbonate-buffered basal salt medium amended with 10 μL isoprene (~100.0 μmol), 20 mL H_2_, and Wolin vitamins. For energy metabolism analysis, strain Y cultures were grown under three different conditions: (i) isoprene-amended cultures with 20.0 mL H_2_ (ISO-1), (ii) cultures with 20.0 mL H_2_ without isoprene (H_2_ plus CO_2_), and (iii) cultures with isoprene and 22.4 mL H_2_ (ISO-2). Samples collected from the first two growth conditions were subjected to proteomic analysis. To investigate if isoprene transformation was HCO_3_^−^/CO_2_ and/or H_2_ dependent, the buffer system was replaced with 4-(2-hydroxyethyl)-1-piperazineethanesulfonic acid (HEPES; 20 mM, pH 7.3), and 100% N_2_ was provided in the headspace. To test hydrogenation of other compounds with at least one C=C bond, isoprene was replaced with 2 mL ethene (~0.14 mM in the liquid phase), 2 mL propene (~0.14 mM), 10 mM fumarate, or 2.5 mL 1,3-butadiene (~0.35 mM).

### DNA extraction, amplicon sequencing and Sanger sequencing.

Genomic DNA was extracted from ~0.5 g sediment slurry or 1 mL of culture suspension using the TIANamp soil DNA kit (Tiangen Biotech, Beijing, China) following the manufacturer’s instructions. For amplicon sequencing, the hypervariable V3-V4 region of the bacterial 16S rRNA gene was amplified using the primers V3-V4-F and V3-V4-R (Table S2) (39). Sequencing was performed by GENEWIZ Inc. (Tianjin, China) using an Illumina MiSeq PE250/300 platform (40). In brief, DNA libraries were prepared using 20 to 30 ng DNA as the template with the MetaVx library preparation kit (GENEWIZ Inc., South Plainfield, NJ, USA). The multiplexed DNA libraries were sequenced with an Illumina MiSeq instrument following the manufacturer's instructions (Illumina, San Diego, CA, USA). Base calling and image analysis were performed using the embedded MiSeq Control software with default parameters. Raw sequencing reads were paired and analyzed using the mothur software package (www.mothur.org) following MiSeq standard operating procedures (41). Quality-controlled and trimmed sequences were uploaded into the SILVAngs server for comparison analysis with default parameters (42). Sequences were grouped into operational taxonomic units at a similarity threshold of 97%. For Sanger sequencing, near-full-length bacterial 16S rRNA genes were amplified with general primers 27F and 1492R (Table S2) using a Veriti 96-well thermal cycler (Thermo Fisher Scientific, Waltham, MA, USA) as described elsewhere (43). PCR products were visualized on 1% agarose gels and purified using an UltraClean 15 DNA purification kit (MoBio, Inc., Carlsbad, CA, USA), prior to Sanger sequencing performed by GENEWIZ Inc.

10.1128/mbio.02086-22.9 TABLE S2 Nucleotide sequences of the primers and probe used in the PCR and qPCR assays targeting bacterial 16S rRNA genes. Download Table S2, DOCX file, 0.03 MB. Copyright © 2022 Jin et al. 2022 Jin et al. https://creativecommons.org/licenses/by/4.0/ This content is distributed under the terms of the Creative Commons Attribution 4.0 International license.

### Quantitative PCR.

TaqMan chemistry-based quantitative PCR (qPCR) primer set, Aceto-786F (5′-GGTAGTCCACGCCGTAAACG-3′) and Aceto-866R (5′-CAGGCGGAGTGCTTATTGC-3′), and probe Aceto-829 probe (5′–6-carboxyfluorescein–CTCAGTGCCGCAGCT–MGB–3′) targeting the 16S rRNA gene of strain Y were designed using Primer3Plus (Whitehead Institute for Biomedical Research, Cambridge, USA) (44). Primer specificity and self-complementarity were verified using Primer-BLAST software (NCBI) and Oligo Calculator version 3.27 (Northwestern University, Chicago, USA) (45). Each qPCR mixture (25 μL) contained 12.5 μL of 2× Premix Ex Taq master mix (TaKaRa Bio Inc., Beijing, China), 0.5 μL of 50× ROX reference dye II, 0.5 μL of each primer (0.2 μM final concentration), 1 μL of probe (0.4 μM final concentration), 2 μL of DNA template, and 8 μL nuclease-free water. The thermocycling program was as follows: 50°C for 2 min and then held at 95°C for 10 min, followed by 40 cycles of 15 s at 95°C and 1 min at 60°C. All qPCR assays were performed on a QuantStudio 3 real-time PCR system (Applied Biosystems, Waltham, MA, USA). Calibration curves were generated using independently diluted plasmid DNA standards containing a partial 16S rRNA gene fragment of strain Y. The partial 16S rRNA gene fragment of strain Y was PCR amplified using the primer set Aceto-737F and Aceto-1177R (Table S2). The 440-bp amplicons were cloned into a pESI-T vector using a TOPO-TA cloning kit (Invitrogen, Carlsbad, CA, USA). The plasmids were extracted from the Escherichia coli clone and used for generating qPCR standard. The qPCR assay exhibited an amplification efficiency of 97.8%, a detection limit of 2.6 × 10^2^ gene copies per reaction, and a linear range of 2.6 × 10^3^ to 2.6 × 10^10^ gene copies per reaction (Fig. S7).

10.1128/mbio.02086-22.7 FIG S7 Amplification plot (A) and standard curve (B) of the qPCR assay targeting the 16S rRNA gene of *A. wieringae* strain Y. Plasmid DNA containing a 16S rRNA gene fragment of strain Y was diluted to obtain gene copy numbers ranging from 2.61 × 10^3^ to 2.61 × 10^10^. All qPCR reactions were performed in triplicate. Download FIG S7, TIF file, 1.4 MB. Copyright © 2022 Jin et al. 2022 Jin et al. https://creativecommons.org/licenses/by/4.0/ This content is distributed under the terms of the Creative Commons Attribution 4.0 International license.

### Genome sequencing, assembly, and annotation.

Cells were harvested in the stationary phase by centrifugation at 13,000 × *g* for 30 min at 4°C. Genomic DNA was extracted using the sodium dodecyl sulfate (SDS) method (46). Whole-genome sequencing was performed by Novogene Bioinformatics Technology Co., Ltd. (Beijing, China) using a combined PacBio (PacBio, Menlo Park, CA, USA) and Illumina NovaSeq PE150 (Illumina Inc., San Diego, CA, USA) sequencing strategy. The long-insert library for PacBio sequencing was constructed with an insert size of ~10 kb using the single-molecule real-time bell template kit (Pacific Biosciences) following the manufacturer’s instructions. For Illumina sequencing, the library with an average insert size of 350 bp was constructed using the NEBNext Ultra DNA library prep kit (New England Biolabs, Ipswich, MA, USA) according to the manufacturer’s recommendations. The size distribution of the insert fragment was analyzed using an Agilent 2100 Bioanalyzer (Agilent Technologies, Santa Clara, CA, USA). The genome was assembled from PacBio long reads and Illumina short reads using Unicycler version 0.47 with default parameters (47). The complete genome of strain Y was visualized with CGView server (GC-skew). The National Center for Biotechnology Information (NCBI) Prokaryotic Genome Annotation Pipeline was used for open reading frame prediction and functional annotation (48).

### Scanning electron microscopy.

For immersion fixation, freshly harvested strain Y cell pellets were suspended in 2.5% glutaraldehyde buffer at 4°C for 4 h, followed by centrifuging the suspension at 14,000 × *g* for 15 min. The treated pellets were rinsed gently with 100 mM phosphate-buffered saline (PBS) solution at pH 7.3 three times and then incubated with 1% (wt/vol) osmic acid for 2 h at room temperature. Cells were washed again with PBS and then dehydrated through a series of 30%, 50%, 70%, 85%, 95%, and 100% ethanol for 10 min each. Cell samples were immersed with *tert*-butanol and transferred into a glass flat plate coated with aluminum film. After overnight lyophilization in a freeze dryer (Millrock Tech, Kingston, NY, USA), the samples were scanned and imaged using an FEI Inspect F50 field emission electron microscope (FEI Company, Mahwah, NJ, USA) under high-performance conditions with accelerating voltages reaching 20 kV.

### Protein extraction and labeling.

Cells in the exponential phase were collected from triplicate isoprene-fed strain Y cultures and triplicate control cultures without an isoprene addition. Cell pellets were rinsed twice with 10 mM cold PBS and then suspended in 200 μL pH 8.0 lysis buffer (100 mM dithiothreitol, 4% SDS, and 150 mM Tris-HCl). The six samples were ultrasonicated in a boiling water bath for 10 min. Cellular debris was removed by centrifugation at 13,000 × *g* for 15 min. The supernatant was quantified with a bicinchoninic acid protein assay kit (GLPBIO, Montclair, CA, USA) according to the manufacturer’s instructions. SDS-PAGE (8 to 16%) for total crude proteins was used to perform a preliminary difference assessment. Protein digestion was performed following the previously described filter-aided sample preparation method (49). Peptide concentrations were estimated using a Nanodrop 2000c spectrophotometer (Thermo Fisher Scientific). Each 100 μg of peptide was labeled with equivalent Tandem Mass Tag (TMT) reagents according to the manufacturer’s instructions (Thermo Fisher Scientific) (50). The TMT-labeled peptides were separated using a Pierce high-pH reversed-phase peptide fractionation kit (Thermo Fisher Scientific). A total of 30 fractions were collected and merged into 10 components. Eventually, these fractions were dried and dissolved in 0.1% formic acid solution for subsequent nano-liquid chromatography coupled with tandem mass spectrometry (LC–MS/MS) analysis.

### Nano-LC-MS/MS analysis.

LC-MS analysis of peptides was conducted using a Q-Exactive mass spectrometer system coupled to an Easy nLC 1200 HPLC system (Thermo Fisher Scientific, Waltham, MA, USA). Samples were injected into a C_18_ trap precolumn (100 μm by 20 mm, 5 μm, Dr. Maisch GmbH, Ammerbuch, Germany) and separated using a C_18_ analytical column (75 μm by 150 mm, 3 μm, Dr. Maisch GmbH) at a flow rate of 300 nL min^−1^. The mobile phases consisted of deionized water with 0.1% formic acid (solution A) and 95% acetonitrile with 0.1% formic acid (solution B). The elution procedure was as follows: the fraction of solution B increased from 2% to 8% during the initial 2 min, increased to 30% over the next 40 min, further to 45% over 8 min, rapidly to 100% over an additional 1-min time period, and then held at 100% for 10 min. MS1 and MS2 spectra based on the higher-energy collisional dissociation (HCD) method were acquired in the Orbitrap in positive ionization mode at resolutions of 60,000 and 1,500, respectively. MS spectra of the full scan were acquired over a range of 350 to 2,000 *m/z*. The top 20 abundant precursor ions were selected for HCD fragmentation. The normalized collision energy was 32 eV. Raw data were retrieved using a search engine (Sequest HT) with default parameters in Proteome Discoverer 2.4 software (version 1.6.0.16) for protein identification against strain Y genome. The false-discovery rate (FDR) was set to 1%, and TMT reporter ion intensity was used for quantification. Expression data were grouped together by hierarchical clustering according to the protein level. Protein functional annotation was conducted using the Universal Protein (UniProt) database (51).

### Analytical methods.

Unsaturated hydrocarbons were measured by injecting 100 μL headspace gas into an Agilent 7890A gas chromatograph equipped with a flame ionization detector and an Agilent DB-624 column as described elsewhere (52). The hydrogenation rate of isoprene was calculated based on the production of methyl-1-butenes (2-methyl-1-butene and 3-methyl-1-butene) during a linear transformation range represented by at least three measurements. Acetate, fumarate, and succinate were analyzed using an Agilent 1260 high-performance liquid chromatography (HPLC) system (Santa Clara, CA, USA) equipped with an Aminex HPX-87H column (Bio-Rad, Hercules, CA, USA) and a diode-array detector set at 210 nm as described elsewhere (53). Aqueous samples were passed through 0.22-μm HPLC-grade syringe filters (Pall Life Sciences, England) and acidified with 0.1% (vol/vol; i.e., 18.8 mM) H_2_SO_4_. Undiluted or diluted samples were separated at a flow rate of 0.6 mL min^−1^ using 4 mM H_2_SO_4_ as the mobile phase.

### Phylogenetic analysis.

Neighbor-joining phylogenetic tree estimation of selected 16S rRNA genes was performed using Geneious Prime 2020.2.4 with 1,000 bootstrap replicates. Evolutionary model selection was evaluated with Tamura-Nei (54). The result was exported as a Newick tree and uploaded into the Interactive Tree of Life (iTOL) server for visualization and annotation (https://itol.embl.de) (55). The maximum-likelihood distance trees of (putative) ene-reductase proteins were built using the Clustal W alignment (56) and Mega 11.0.10 software (57) with default settings. The GenBank accession numbers and detailed information of the protein sequences are listed in Data Set S2.

### Data availability.

The complete genome sequence of strain Y has been deposited in GenBank under accession number CP087994. The BioSample and BioProject accession numbers are SAMN23007773 and PRJNA778957, respectively. Raw sequencing reads have been deposited in the Sequence Read Archive under the accession numbers SRR18087747 (Illumina) and SRR18091988 (PacBio) for the strain Y genome and accession number PRJNA808885 for the 16S rRNA gene amplicon sequencing reads. Data Sets S1 to S4 are available through the Figshare platform and can be accessed via https://doi.org/10.6084/m9.figshare.20349294.v2.
