# Supplementary figures and images for: Anaerobic Biohydrogenation of Isoprene by Acetobacterium wieringae Strain Y
Source: mBio. 2022 Nov 7;13(6):e02086-22. doi: 10.1128/mbio.02086-22 (PMC9765523; doi:10.1128/mbio.02086-22)

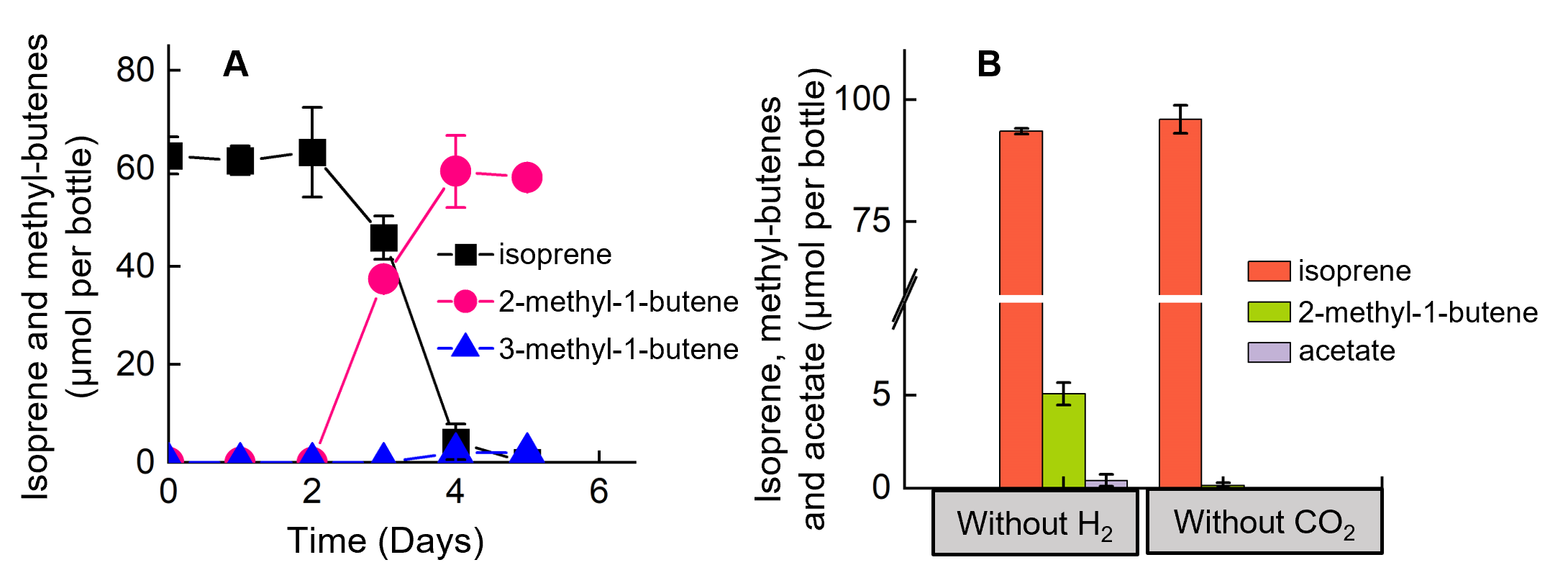

Supplement: FIG S1 [file mbio.02086-22-s0002.tif]

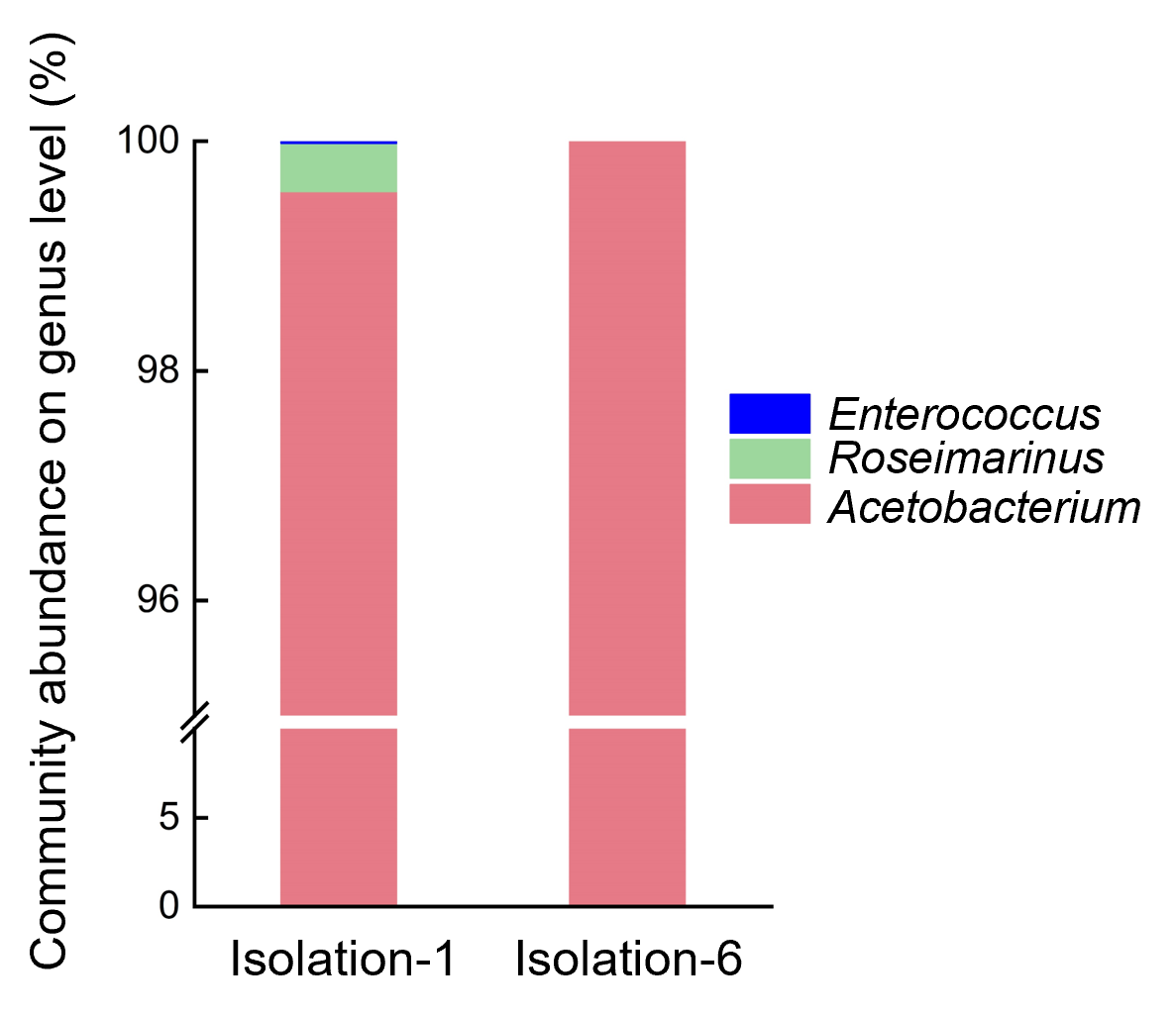

Supplement: FIG S2 [file mbio.02086-22-s0003.tif]

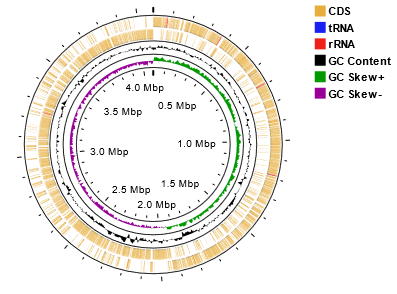

Supplement: FIG S3 [file mbio.02086-22-s0004.tif]

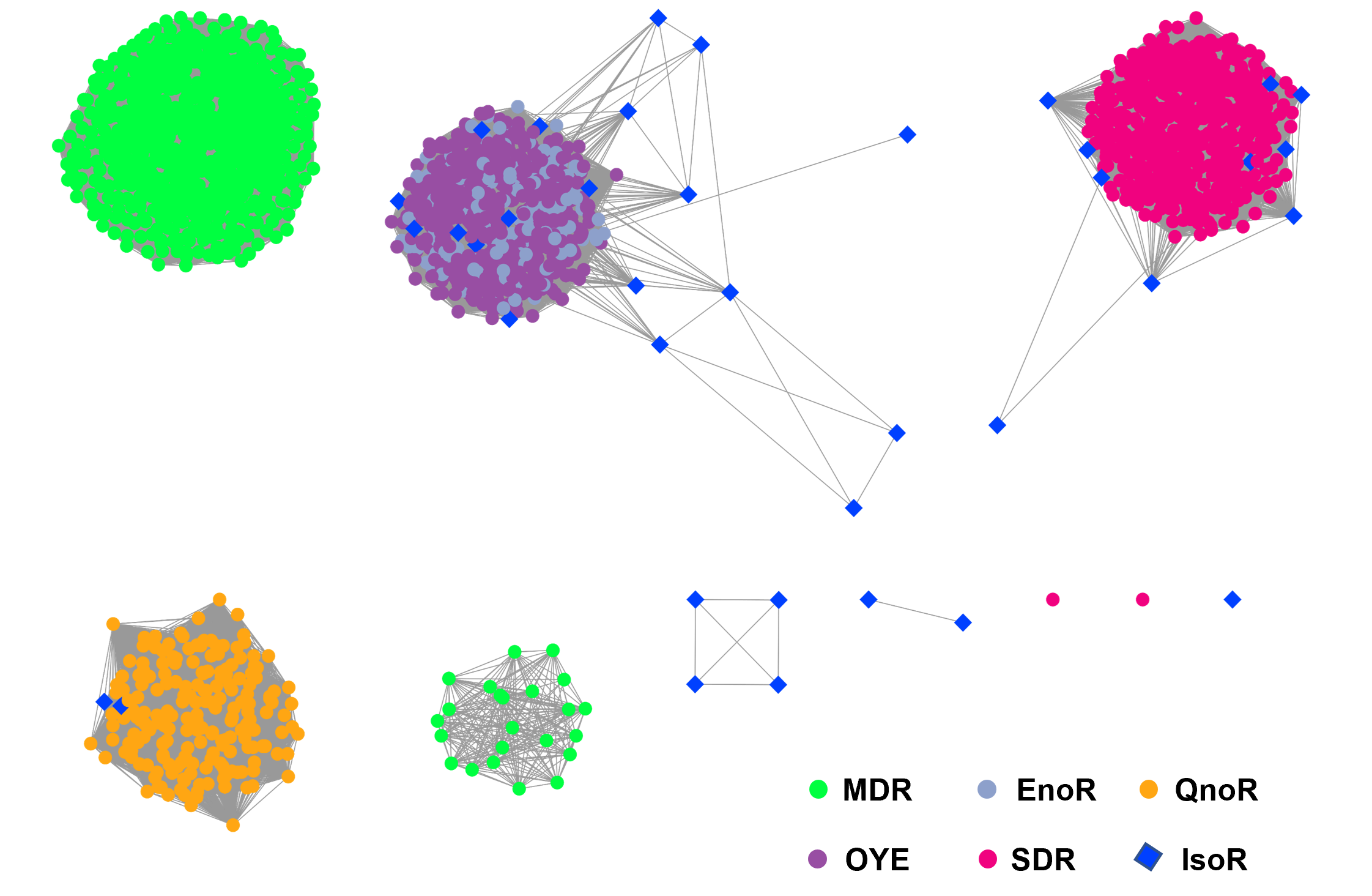

Supplement: FIG S4 [file mbio.02086-22-s0005.tif]

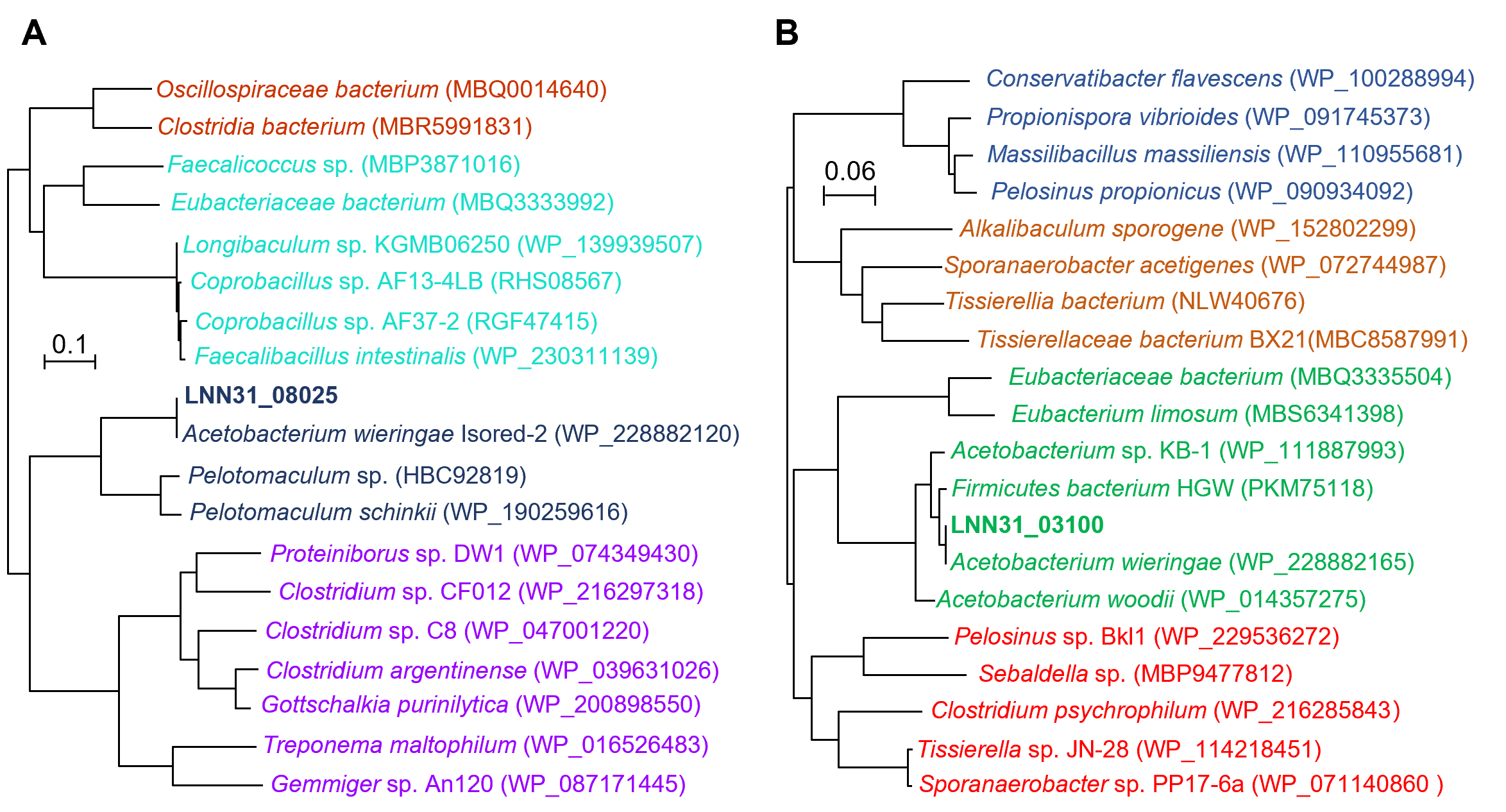

Supplement: FIG S5 [file mbio.02086-22-s0006.tif]

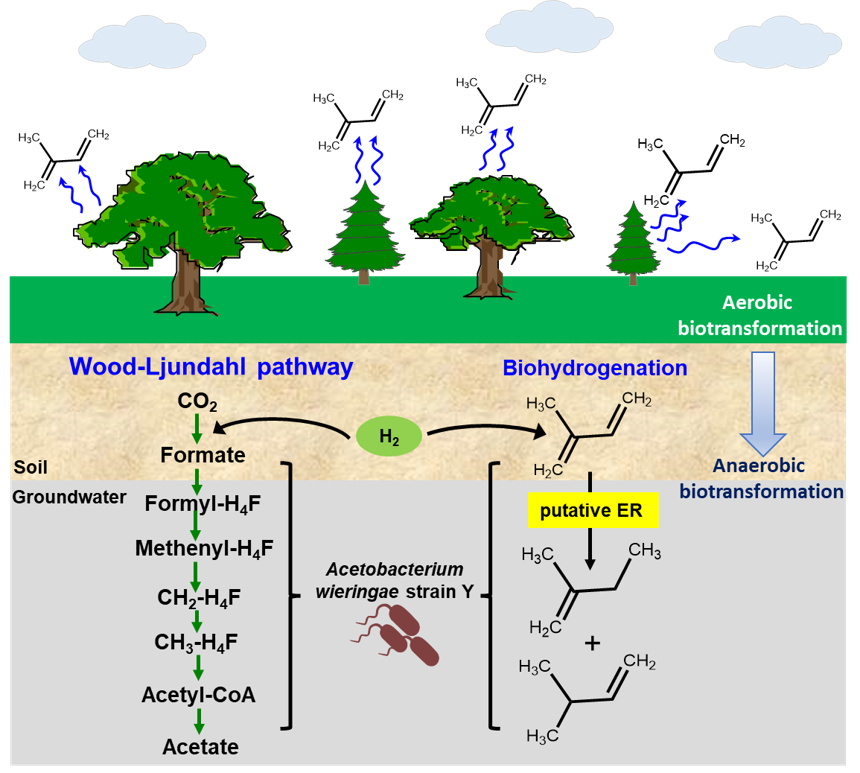

Supplement: FIG S6 [file mbio.02086-22-s0007.tif]

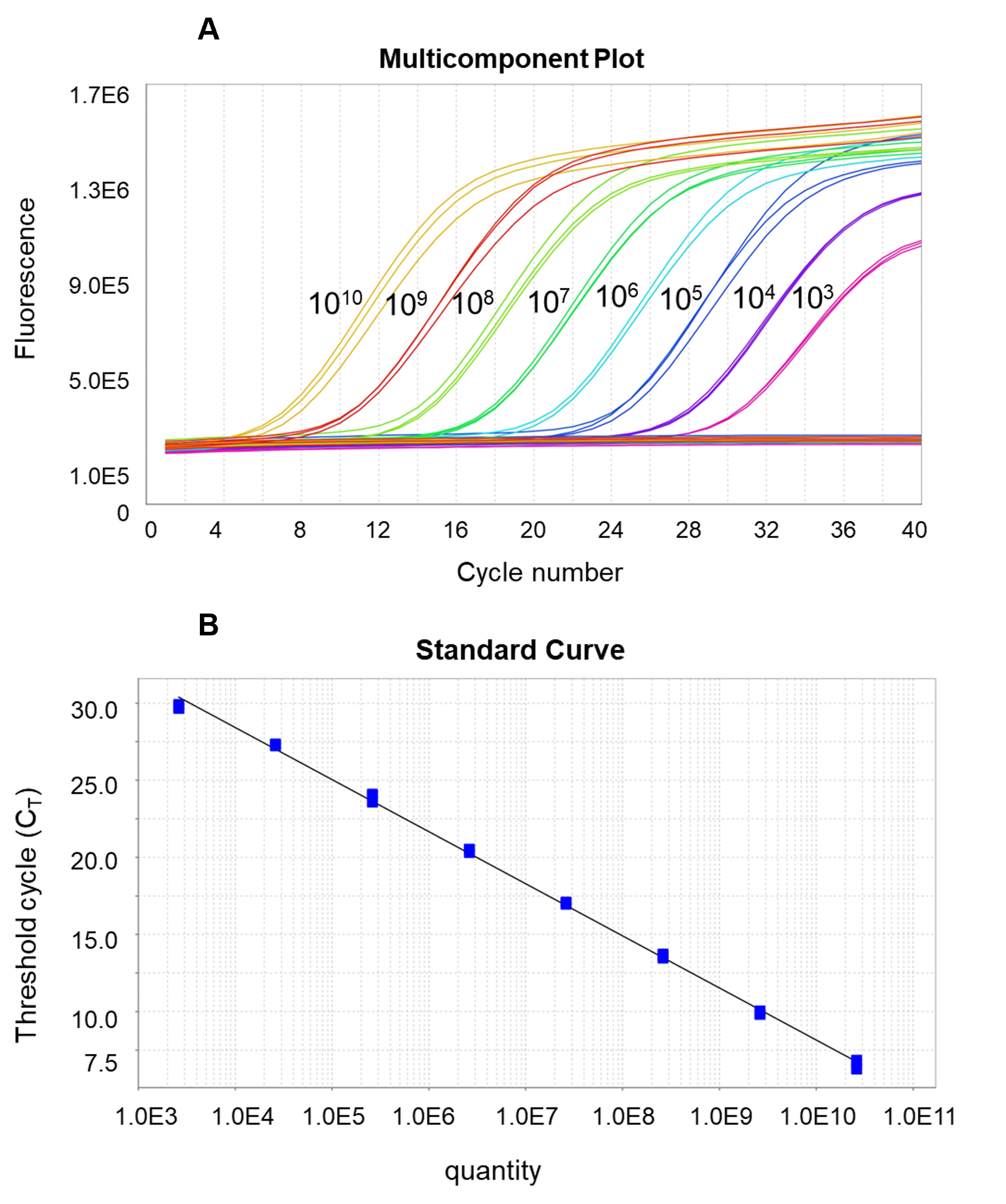

Supplement: FIG S7 [file mbio.02086-22-s0008.tif]
