# Supplementary material for: Anaerobic Biohydrogenation of Isoprene by Acetobacterium wieringae Strain Y
Source: mBio. 2022 Nov 7;13(6):e02086-22. doi: 10.1128/mbio.02086-22 (PMC9765523; doi:10.1128/mbio.02086-22)
Supplement: TEXT S1 [file mbio.02086-22-s0001.docx]

**Anaerobic** **biohydrogenation of isoprene by *Acetobacterium wieringae* strain Y**

Huijuan Jin^1,2^, Xiuying Li^1^, Hongyan Wang^1,2^, Natalie L. Cápiro^3^, Xiaocui Li^1,2^, Frank E. Löffler ^4-8^, Jun Yan^1^, Yi Yang^1*^

^1^Key Laboratory of Pollution Ecology and Environmental Engineering, Institute of Applied Ecology, Chinese Academy of Sciences; Shenyang, Liaoning 110016, China; ^2^ University of Chinese Academy of Sciences, Beijing, China, 100049; ^3^Department of Civil and Environmental Engineering, Auburn University, Auburn, AL 36849, USA; ^4^Center for Environmental Biotechnology, ^5^Department of Civil and Environmental Engineering, ^6^Department of Microbiology, and ^7^Department of Biosystems Engineering & Soil Science, University of Tennessee, Knoxville, Tennessee 37996, USA; ^8^Biosciences Division, Oak Ridge National Laboratory, Oak Ridge, Tennessee 37831, USA

* Corresponding authors

Yi Yang

Key Laboratory of Pollution Ecology and Environmental Engineering

Institute of Applied Ecology, Chinese Academy of Sciences

Shenyang, Liaoning 110016, China

Phone: +86-24-83970426

E-mail: [yangyi@iae.ac.cn](mailto:yangyi@iae.ac.cn)

**This supplementary file contains:** Supplementary Methods.

Supplementary Information Text

**Comparative genomic analysis.** Strain Y genome along with 14 publicly available *Acetobacterium* genomes retrieved from the GenBank were used for comparative analysis. The DNA-DNA hybridization (DDH) values were calculated with the web Genome-to-Genome Distance Calculator 3.0 (GGDC, http://ggdc.dsmz.de/ggdc.php#) using strain Y genome as the input sequence (1). The average nucleotide identity (ANIm, ANIb, and orthoANI) values between strain Y genome and the each *Acetobacterium* genome were calculated using the web server JSpecies 3.9.0 (http://jspecies.ribohost.com/jspeciesws/) (2) and CJ Bioscience's online ANI Calculator (https://www.ezbiocloud.net/tools/ani) (3).

***In silico* identification of putative ene reductases.** The BLASTP search resulted in the identification of twenty-one putative ene-reductase enzymes including nine NAD(P)/FAD-dependent oxidoreductase belonging to OYE or EnoR families (sequence identity ≥ 27.4%, coverage ≥ 91%), ten NAD(P)-dependent oxidoreductases belonging to SDR family (sequence identity ≥ 19.0%, coverage ≥ 30%), and two flavin reductase proteins belonging to QnoR family (sequence identity ≥ 69.3%, coverage ≥ 73%). In addition to the above twenty-one putative ene-reductases, another twenty-three NAD(P)H/FAD-dependent oxidoreductases sequences were searched and inspected manually (Dataset S1).

**Construction of sequence similarity network (SSN).** A total of 2,905 characterized or putative ene reductase sequences were extracted from the KEGG database with known EC numbers including EC 1.6.99.1 (OYE family of oxidoreductases), EC 1.3.1.48 and EC 1.3.1.74 (medium-chain dehydrogenases/reductases, MDR), EC 1.3.1.31 (Fe-S cluster containing enoate reductases, EnoR), and EC 1.1.1.208 (short-chain dehydrogenase/reductase salutaridine/menthone reductase-like subfamily, SDR). The recently characterized two quinone reductases-like ene reductases (QnoR) belonging to a novel ER subfamily and their homologs were retrieved from the NCBI database by BLAST search against NCBI non-redundant database with accession numbers WP_010884948.1 and WP_011173882.1 (4). Sequence similarity network (SSN) analysis was performed using the Enzyme Function Initiative (EFI-EST) suit hosted on the website (https://efi.igb.illinois.edu/efi-est/) (5). The network was then generated with an alignment score threshold of 10^-10^, resulting a total of 4,422 nodes and 2,673,538 edges. Cytoscape 3.9.1 (6) was used for visualizing the generated representative node networks.

**SI References**

1. Meier-Kolthoff JP, Carbasse JS, Peinado-Olarte RL, Göker M. 2022. TYGS and LPSN: a database tandem for fast and reliable genome-based classification and nomenclature of prokaryotes. *Nucleic Acids Res* 50:D801-D807. <https://doi.org/10.1093/nar/gkab902>.

2. Richter M, Rosselló-Móra R, Oliver Glöckner F, Peplies J. 2016. JSpeciesWS: a web server for prokaryotic species circumscription based on pairwise genome comparison. *Bioinformatics* 32:929-931. <https://doi.org/10.1093/bioinformatics/btv681>.

3. Yoon S-H, Ha S-M, Lim J, Kwon S, Chun J. 2017. A large-scale evaluation of algorithms to calculate average nucleotide identity. *Antonie Van Leeuwenhoek* 110:1281-1286. <https://doi.org/10.1007/s10482-017-0844-4>.

4. Steinkellner G, Gruber CC, Pavkov-Keller T, Binter A, Steiner K, Winkler C, Łyskowski A, Schwamberger O, Oberer M, Schwab H. 2014. Identification of promiscuous ene-reductase activity by mining structural databases using active site constellations. *Nat Commun* 5:1-9. <https://doi.org/10.1038/ncomms5150>.

5. Zallot R, Oberg N, Gerlt JA. 2019. The EFI web resource for genomic enzymology tools: leveraging protein, genome, and metagenome databases to discover novel enzymes and metabolic pathways. *Biochemistry* 58:4169-4182. <https://doi.org/10.1021/acs.biochem.9b00735>.

6. Shannon P, Markiel A, Ozier O, Baliga NS, Wang JT, Ramage D, Amin N, Schwikowski B, Ideker T. 2003. Cytoscape: a software environment for integrated models of biomolecular interaction networks. *Genome Res* 13:2498-2504. <https://doi.org/10.1101/gr.1239303>.

7. Balch WE, Magrum LJ, Fox GE, Wolfe RS, Woese CR. 1977. An ancient divergence among the bacteria. *J Mol Evol* 9:305-311. <https://doi.org/10.1007/BF01796092>.

8. Westphal L, Wiechmann A, Baker J, Minton NP, Müller V. 2018. The Rnf complex is an energy-coupled transhydrogenase essential to reversibly link cellular NADH and ferredoxin pools in the acetogen *Acetobacterium woodii*. *J Bacteriol* 200:e00357-18. <https://doi.org/10.1128/JB.00357-18>.

9. Methé BA, Nelson KE, Deming JW, Momen B, Melamud E, Zhang X, Moult J, Madupu R, Nelson WC, Dodson RJ. 2005. The psychrophilic lifestyle as revealed by the genome sequence of *Colwellia psychrerythraea* 34H through genomic and proteomic analyses. *Proc Natl Acad Sci U S A* 102:10913-10918. <https://doi.org/10.1073/pnas.0504766102>.

10. Bellion E, Tan F. 1987. An NAD^+^-dependent alanine dehydrogenase from a methylotrophic bacterium. *Biochem J* 244:565-570. <https://doi.org/10.1042/bj2440565>.

11. Ashida H, Saito Y, Kojima C, Kobayashi K, Ogasawara N, Yokota A. 2003. A functional link between RuBisCO-like protein of *Bacillus* and photosynthetic RuBisCO. *Science* 302:286-290. <https://doi.org/10.1126/science.1086997>.

12. Kavanagh K, Jörnvall H, Persson B, Oppermann U. 2008. Medium-and short-chain dehydrogenase/reductase gene and protein families. *Cell Mol Life Sci* 65:3895-3906. <https://doi.org/10.1007/s00018-008-8590-4>.

13. Valentini M, Filloux A. 2016. Biofilms and cyclic di-GMP (c-di-GMP) signaling: lessons from *Pseudomonas aeruginosa* and other bacteria. *J Biol Chem* 291:12547-12555. <https://doi.org/10.1074/jbc.R115.711507>.

14. Hess V, Schuchmann K, Müller V. 2013. The ferredoxin: NAD^+^ oxidoreductase (Rnf) from the acetogen *Acetobacterium woodii* requires Na^+^ and is reversibly coupled to the membrane potential. *J Biol Chem* 288:31496-31502. <https://doi.org/10.1074/jbc.M113.510255>.

15. Ross DE, Marshall CW, Gulliver D, May HD, Norman RS. 2020. Defining genomic and predicted metabolic features of the *Acetobacterium* genus. *mSystems* 5:e00277-20. <https://doi.org/10.1128/mSystems.00277-20>.

16. Grostern A, Edwards EA. 2009. Characterization of a *Dehalobacter* coculture that dechlorinates 1,2-dichloroethane to ethene and identification of the putative reductive dehalogenase gene. *Appl Environ Microbiol* 75:2684-2693. <https://doi.org/10.1128/AEM.02037-08>.

17. Badger MR, Bek EJ. 2008. Multiple Rubisco forms in proteobacteria: their functional significance in relation to CO_2_ acquisition by the CBB cycle. *J Exp Bot* 59:1525-1541. <https://doi.org/10.1093/jxb/erm297>.

18. Fernandez NL, Waters CM. 2019. Cyclic di-GMP increases catalase production and hydrogen peroxide tolerance in *Vibrio cholerae*. *Appl Environ Microbiol* 85:e01043-19. <https://doi.org/10.1128/AEM.01043-19>.

19. Frank JA, Reich CI, Sharma S, Weisbaum JS, Wilson BA, Olsen GJ. 2008. Critical evaluation of two primers commonly used for amplification of bacterial 16S rRNA genes. *Appl Environ Microbiol* 74:2461-2470. <https://doi.org/10.1128/AEM.02272-07>.

20. Teng F, Nair SSD, Zhu P, Li S, Huang S, Li X, Xu J, Yang F. 2018. Impact of DNA extraction method and targeted 16S rRNA hypervariable region on oral microbiota profiling. *Sci Rep* 8:1-12. <https://doi.org/10.1038/s41598-018-34294-x>.
