# Supplementary material for: Anaerobic Biohydrogenation of Isoprene by Acetobacterium wieringae Strain Y
Source: mBio. 2022 Nov 7;13(6):e02086-22. doi: 10.1128/mbio.02086-22 (PMC9765523; doi:10.1128/mbio.02086-22)
Supplement: TABLE S1 [file mbio.02086-22-s0009.docx]

**TABLE S1** Genome statistics of 15 *Acetobacterium* strains (See references in Supplementary Information).

| **Strain** | **GenBank accession** **no.** | **Genome** | **G+C** | | **dDDH** | | | **ANI (%)** | | | | **References** |
| --- | --- | --- | --- | --- | --- | --- | --- | --- | --- | --- | --- | --- |
|  |  | (Mb) | (%) | | (%) | OrthoAN | | | | ANIm | ANIb |  |
| *A. wieringae* strain Y | CP087994 | 4.16 | 44.2 | 100 | | | 100 | | 100 | | 100 | - |
| *A. woodii* DSM 1030 | CP002987 | 4.04 | 39.3 | 21.5 | | | 77.8 | | 84.0 | | 76.2 | (7, 8) |
| *A. bakii* DSM 8239 | LGYO00000000 | 4.14 | 41.2 | 20.2 | | | 74.4 | | 84.0 | | 73.6 | (9, 10) |
| *A. dehalogenans* DSM 11527 | AXAC00000000 | 4.05 | 43.8 | 27.5 | | | 83.5 | | 86.1 | | 82.7 | (11) |
| *A. malicum* DSM 4132 | MJUY00000000 | 4.09 | 43.7 | 27.5 | | | 83.3 | | 86.1 | | 82.6 | (12) |
| *A. paludosum* DER-2019 | WJBD00000000 | 3.70 | 40.1 | 19.9 | | | 74.2 | | 83.3 | | 73.7 | (9) |
| *A. tundrae* DER-2019 | WJBB00000000 | 3.57 | 39.7 | 19.7 | | | 74.4 | | 83.7 | | 73.8 | (13) |
| *A. wieringae* DSM 1911 | LKEU00000000 | 3.90 | 44.1 | 73.5 | | | 97.4 | | 97.6 | | 96.7 | (14) |
| *A. fimetarium* DSM 8238 | WJBC00000000 | 3.25 | 44.7 | 20.5 | | | 74.6 | | 84.9 | | 74.4 | (9) |
| *Acetobacterium* sp. KB-1 | CP030040 | 3.98 | 42.5 | 24.6 | | | 80.6 | | 84.7 | | 80.3 | (15, 16) |
| *Acetobacterium* sp. MES1 | AXAC00000000 | 3.65 | 44.3 | 76.6 | | | 97.4 | | 97.5 | | 96.5 | (17) |
| *Acetobacterium* sp. UBA6819 | DKEP00000000 | 3.61 | 42.8 | 24.2 | | | 80.8 | | 84.5 | | 80.2 | (18) |
| *Acetobacterium* sp. UBA5558 | DIMU00000000 | 2.61 | 43.6 | 77.1 | | | 97.6 | | 97.6 | | 97.1 | (18) |
| *Acetobacterium* sp. UBA11218 | DNHO00000000 | 2.22 | 44.8 | 64.9 | | | 95.9 | | 95.3 | | 95.6 | (18) |
| *Acetobacterium* sp. UBA5834 | DJGY00000000 | 3.46 | 44.1 | 64.8 | | | 97.3 | | 97.4 | | 96.8 | (18) |
