# Supplementary material for: Anaerobic Biohydrogenation of Isoprene by Acetobacterium wieringae Strain Y
Source: mBio. 2022 Nov 7;13(6):e02086-22. doi: 10.1128/mbio.02086-22 (PMC9765523; doi:10.1128/mbio.02086-22)
Supplement: TABLE S2 [file mbio.02086-22-s0010.docx]

**TABLE S2** Nucleotide sequences of the primers and probe used in the PCR and qPCR assays targeting bacterial 16S rRNA genes (See references in Supplementary Information).

| **Primer** | **Sequence (5’-3’)** | **Target gene** | **Reference** | |  |
| --- | --- | --- | --- | --- | --- |
| 27F | AGAGTTTGATCCTGGCTCAG | Bacterial 16S rRNA | | (19) | |
| 1492R | GGTTACCTTGTTACGACTT |  |  |  |  |
| V3-V4-F | CCTACGGRRBGCASCAGKVRVGAAT ^a^ | Bacterial 16S rRNA | | (20) | |
| V3-V4-R | GGACTACNVGGGTWTCTAATCC ^a^ |  |  |  |  |
| Aceto-737F | ACTGACGCTGAGGTGCGAAAGC | *A. wieringae* strain Y 16S rRNA | | This study | |
| Aceto-1177R | TGACGTCGTCCCCACCTTCC |  |  |  |  |
| Aceto-786F | GGTAGTCCACGCCGTAAACG | *A. wieringae* strain Y 16S rRNA | | This study | |
| Aceto-866R | CAGGCGGAGTGCTTATTGC |  |  |  |  |
| Aceto-829Probe | 6FAM-CTCAGTGCCGCAGCT-MGB ^b^ |  |  |  |  |

^a^ Degenerate bases, R=A/G, B=C/G/T, S=C/G, K=G/T, V=A/C/G, N=A/C/G/T, W=A/T.

^b^ 6FAM, 6-carboxyfluorescein; MGB, minor groove binder moiety.
